# Phenotypic responses to interspecies competition and commensalism in a naturally-derived microbial co-culture

**DOI:** 10.1038/s41598-017-18630-1

**Published:** 2018-01-10

**Authors:** Nymul Khan, Yukari Maezato, Ryan S. McClure, Colin J. Brislawn, Jennifer M. Mobberley, Nancy Isern, William B. Chrisler, Lye Meng Markillie, Brett M. Barney, Hyun-Seob Song, William C. Nelson, Hans C. Bernstein

**Affiliations:** 1 Biological Sciences Division, Pacific Northwest National Laboratory, Richland, WA USA; 2 Environmental Molecular Sciences Laboratory, Pacific Northwest National Laboratory, Richland, Washington, USA; 3 Department of Bioproducts and Biosystems Engineering, University of Minnesota, St. Paul, MN 55108 USA; 4 The Gene and Linda Voiland School of Chemical Engineering and Bioengineering, Washington State University, Pullman, WA USA

## Abstract

The fundamental question of whether different microbial species will co-exist or compete in a given environment depends on context, composition and environmental constraints. Model microbial systems can yield some general principles related to this question. In this study we employed a naturally occurring co-culture composed of heterotrophic bacteria, *Halomonas* sp. HL-48 and *Marinobacter* sp. HL-58, to ask two fundamental scientific questions: 1) *how do the phenotypes of two naturally co-existing species respond to partnership as compared to axenic growth?* and 2) *how do growth and molecular phenotypes of these species change with respect to competitive and commensal interactions?* We hypothesized – and confirmed – that co-cultivation under glucose as the sole carbon source would result in competitive interactions. Similarly, when glucose was swapped with xylose, the interactions became commensal because *Marinobacter* HL-58 was supported by metabolites derived from *Halomonas* HL-48. Each species responded to partnership by changing both its growth and molecular phenotype as assayed via batch growth kinetics and global transcriptomics. These phenotypic responses depended on nutrient availability and so the environment ultimately controlled how they responded to each other. This simplified model community revealed that microbial interactions are context-specific and different environmental conditions dictate how interspecies partnerships will unfold.

## Introduction

In microbial life, a cell’s phenotype is determined by the properties encoded in its genome and by its environment. Often, one of the major factors in a microbe’s environment is the presence or absence of other species in the community. When species with the potential to interact come in physical or chemical contact, they respond to each other and often change their phenotypes to enable competition, mutualism, or commensalism. Each of these interaction types represent formative biological principles that have shaped evolution of species and the functioning of entire ecosystems^1,2^. The molecular basis for interspecies microbial interaction is an intense scientific focus area, yet major gaps remain in our collective knowledge of how growth and molecular phenotypes change in response to different modes of microbial partnership. For instance, under one set of environmental constraints two species may compete for a single limiting resource, with the one having greatest affinity for the resource eventually excluding the other. However, natural microbial habitats are rarely static. Conditions that promote competitive exclusion can shift to allow stable co-existence, for example, causing interdependency of growth between two species^3–5^. We addressed this duality by asking two fundamental questions: 1) *how do the phenotypes of two naturally co-existing species respond to partnership as compared to axenic growth?* and 2) *how do growth and molecular phenotypes of these species change with respect to competitive and commensal interactions?*

To answer these questions, we employed a *‘natural’* heterotrophic co-culture comprised of two isolates, *Halomonas* sp. HL-48 and *Marinobacter* sp. HL-58. These strains were co-isolated from a MgSO_4_-dominated hypersaline lake and have been shown to co-exist within stable, naturally interactive microbial consortia^6–8^. Both strains are capable of axenic growth on glucose as the sole carbon and electron source^9^. However, only *Halomonas* HL-48 can utilize xylose – an ability conferred by genes encoding for the ABC-type xylose uptake system (*xylFGH*), a putative xylose isomerase (*CY41DRAFT_1942*) and a xylulokinase (*CY41DRAFT_1366*). *Marinobacter* HL-58, on the other hand, lacks xylose transporters and xylose isomerase genes. It does, however, encode genes that enable it to import and assimilate fermentation products from its co-culture partner: acetate (*acetyl-CoA synthetase*; *acs*), formate (*FNT family formate-nitrate transporter and a formate-tetrahydrofolate ligase; CD01DRAFT_0289*), and ethanol (*PQQ-dependent ethanol dehydrogenase*; *queDH*). These complementary metabolic potentials make this co-culture system well-suited for testing phenotypes associated with substrate competition and commensal metabolite exchange.

A cell’s phenotype is its nonheritable physical state^10^. The phenotype of a population or species is specific to a context, and dependent on the presence or absence of other species^11–15^. This study explores the generalizable concept that microbial species respond to one another as part of their environment and that they exert direct and/or indirect regulation over each other’s expression of genome-encoded functions. Growth phenotypes are direct manifestations of molecular phenotypes. Thus, to assess both the response mechanism and outcome of co-culture under different nutrient-controlled interaction states which spawned either competitive or commensal interactions, we measured the specific growth rates of each species and changes in transcript abundance and metabolite accumulation. Our results suggest that environment can change the nature of interspecies interactions, and that community context modulates an individual species’ response to the environment.

## Results

### Growth phenotypes of competition and commensalism

**Figure 1:**
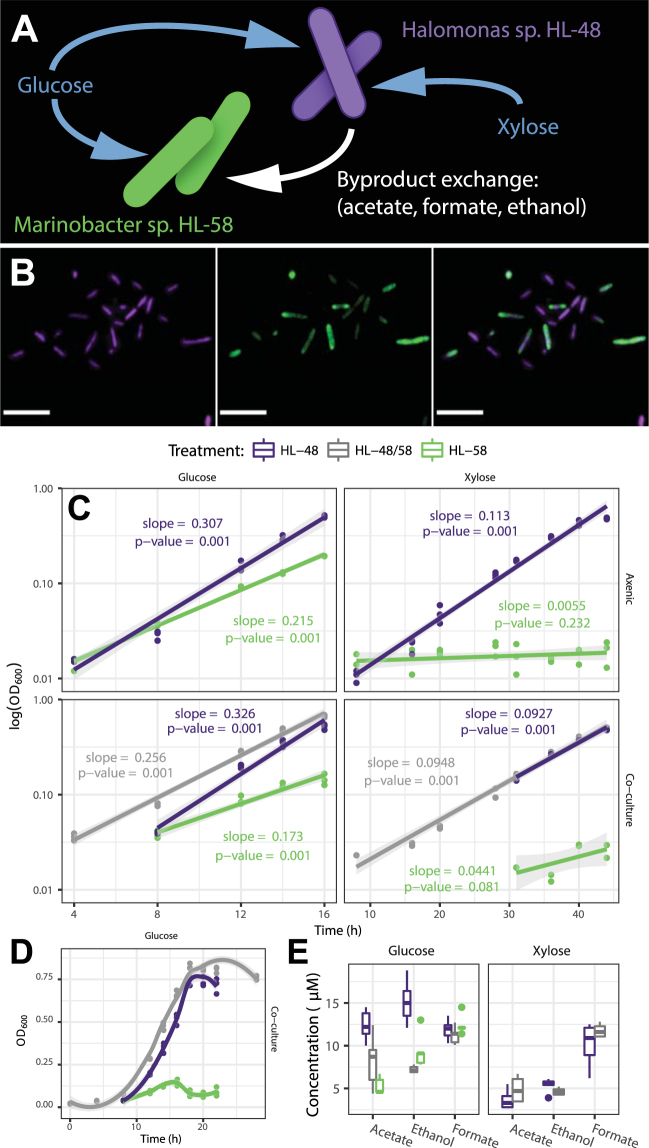
Co-culture of *Halomonas* HL-48 *and Marinobacter* HL-58. (**A**) Both species can metabolize glucose, while only *Halomonas* HL-48 can metabolize xylose. Under xylose co-culture conditions, *Halomonas* HL-48 supports *Marinobacter* HL-58 growth with metabolic byproducts. (**B**) Layers of a confocal image showing the co-existence of each species under xylose (commensal) co-cultivation; scale bars represent 5 µm. A green fluorescent protein was used to quantify the relative populations *Marinobacter* HL-58 (green) against the total (parent) population stained with Alexa Fluor 647 (shown in purple). (**C**) Species-specific growth rates as a function of interspecies partnerships in each nutrient treatment. Data points and regressions that are specific for *Halomonas* HL-48, *Marinobacter* HL-58 and the co-culture are shown in purple, green and grey, respectively. All data points are shown for each replicate measurement. The slopes of each line are equivalent to the specific growth rates (h^−1^) and the p-value represents the probability of observing this slope if there was no relationship between growth rate and time. (**D**) The full growth curves during glucose co-cultivation showing lag through stationary phase; *Marinobacter* HL-58 was outcompeted by *Halomonas* HL-48 as indicated by the drop in abundance and truncation of log-phase growth as compared to the axenic controls. (**E**) Extracellular metabolite concentrations as determined via NMR spectroscopy at the mid-log phase during each treatment (20 h for glucose, 33 h for xylose).

Our co-cultivation experiments demonstrated that interspecies partnerships changed the growth phenotypes of *Halomonas* HL-48 and *Marinobacter* HL-58. These changes depended on whether glucose or xylose was supplied as the sole carbon source. Both species were supported under xylose-fed co-cultivation, indicating that *Marinobacter* HL-58 was present and growing, regardless of the fact that it cannot catabolize xylose as its sole carbon source (Fig. 1A,B,C). Co-cultivation altered the specific growth rate of both species as compared to each respective axenic control (Fig. 1C). The axenic cultures of *Halomonas* HL-48 exhibited higher maximum specific growth rates (0.307 h^−1^) on glucose as compared to axenic *Marinobacter* HL-58 (0.215 h^−1^). Hence, co-cultivation on glucose led to competition, in which growth of *Halomonas* HL-48 outpaced *Marinobacter* HL-58 (Fig. 1D). Under this competitive co-culture treatment, the species displayed contrasting changes in specific growth rates compared to their respective axenic controls (Fig. 1C). *Halomonas* HL-48 exhibited only a 6% increase in growth rate while *Marinobacter* HL-58 was severely affected by competition and decreased its specific growth rate by 20% from its axenic performance. The glucose competing co-culture also showed changes in extracellular organic acid production compared to each axenic control (Fig. 1E). Specifically, competitive co-cultivation resulted in a decreased abundance of acetate and ethanol compared to axenic cultures of *Halomonas* HL-48 (p = 0.02 and p < 0.001, respectively; pairwise t-test with Holm correction).

In contrast to the competitive environment of glucose growth, co-cultivation on xylose established a commensal growth phenotype of the consortium. Growth of *Marinobacter* HL-58 was enabled through partnership with the xylose consuming *Halomonas* HL-48. This result was in stark contrast to the *Marinobacter* HL-58 axenic control on the xylose minimal medium, where no growth could be observed (Fig. 1C). Although *Halomonas* HL-48 exhibited a decreased specific growth rate (18%) in co-cultivation, the duration and general magnitude of log phase growth was essentially identical to axenic performance. Specifically, there was no significant reduction in the overall fitness of *Halomonas* HL-48 as a result of xylose-fed co-cultivation, which is evident by comparing abundances after 46 h of growth (OD_600nm_ = 0.491 ± 0.005 as compared to the axenic measurement of 0.495 ± 0.005). Thus, we conclude that this is an example of commensal behavior rather than parasitism. *Marinobacter* HL-58 growth was confirmed on minimal media containing only acetate and ethanol; hence, it was likely supported in co-culture from alcohol and organic acid metabolic byproducts derived from *Halomonas* HL-48. This conclusion is supported by four pieces of evidence: i) the no-growth axenic phenotype of *Marinobacter* HL-58 on xylose; ii) the absence of xylose uptake/catabolism genes in the *Marinobacter* HL-58 genome; iii) the presence of acetate and ethanol in the extracellular environment (Fig. 1E); and iv) the presence of genes encoding for acetyl-CoA synthetase (*acs*) and alcohol dehydrogenase (*adh* and *queDH*) in the *Marinobacter* HL-58 genome combined with experiments confirming growth on acetate and ethanol provided as the sole carbon source.

### Molecular phenotypes of competition and commensalism

**Figure 2:**
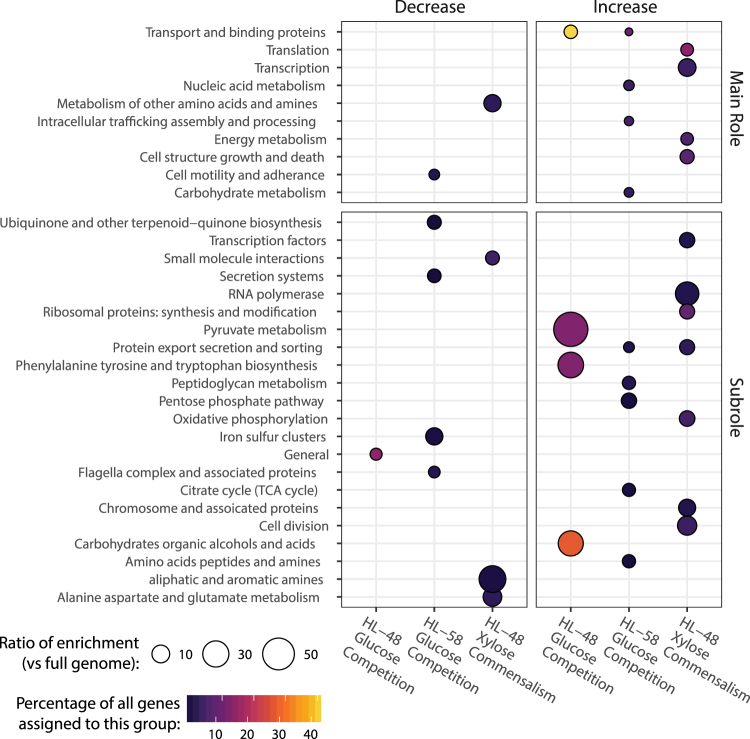
Functional gene categories were differentially expressed under glucose and xylose co-culture relative to axenic culture. Functional enrichment is a measure of the number of differentially expressed genes belonging to a functional category normalized by the total number genes in each species’ genome belonging to the same category. The categories are provided at two levels of detail. The Subroles are more precise functional assignments that are nested within the broader Main Role categories. The functional annotations were derived from DOE-JGI Microbial Genome Annotation and RAST server pipelines. Differential expression was defined as a minimum 2-fold change in abundance and statistical significance (adjusted p-value ≤ 0.05; n = 5). The color scale corresponds to the percentage of differentially expressed genes belonging to each functional group. Higher ratios of enrichment indicate preferential up- or down-regulation of a genome-encoded function in response to competition for glucose or *Halomonas* HL-48 exclusivity for xylose. The ratios, depicted by circle size, represent the percentage of differentially regulated genes belonging to a specific functional module (shown in y-axis) normalized by the percentage of genes assigned to the same function in the genome as a whole.

Distinct molecular phenotypes were observed for each species under the competitive and commensal treatments. This was assayed via global transcriptomic profiles from co-cultivation treatments compared to each respective axenic control. Differentially expressed genes were defined as those showing at least a 2-fold change and adjusted p-value ≤ 0.05 between axenic and co-cultivation. To identify differences in the functional responses of each species between competitive and commensal interactions, we performed functional enrichment analysis on the differentially expressed gene profiles (Fig. 2). The number of differentially expressed genes belonging to each functional category was normalized against the total number genes in each species genome belonging to the same functional category (ratio of enrichment), and this category-level quantitation was compared between treatments.

Differences in gene functions enriched from each species showed distinct molecular phenotypes at the functional level. Competitive co-cultivation resulted in the enrichment of upregulated *Halmonas* HL-48 genes involved in the central carbon metabolism of carbohydrates, alcohols and organic acids as well as genes related to tyrosine and tryptophan biosynthesis. In contrast, *Marinobacter* HL-58 was strongly outcompeted under glucose co-cultivation (Fig. 1C), and populations showed corresponding transcriptional responses by upregulating genes associated with transport proteins, intracellular trafficking as well as core central carbon metabolic functions in the pentose phosphate pathway and TCA cycle. Interestingly, both the *Halomonas* HL-48 and *Marinobacter* HL-58 genomes encode proteins required for glucose catabolism (assigned to the “carbohydrates, organic alcohols and acids” functional category) yet only *Halomonas* HL-48 showed enrichment of this functional category in response to co-culture partnership. The commensal phenotype of the co-culture resulted in *Halomonas* HL-48 upregulating genes associated with transcription, translation, cell structure, growth and death and downregulating certain amino acid and amine metabolism genes. Differential gene expression values corresponding to commensal co-cultivation on xylose could only be obtained for *Halomonas* HL-48 because axenic growth on xylose was not possible for *Marinobacter* HL-58, as designed in this study.

**Figure 3:**
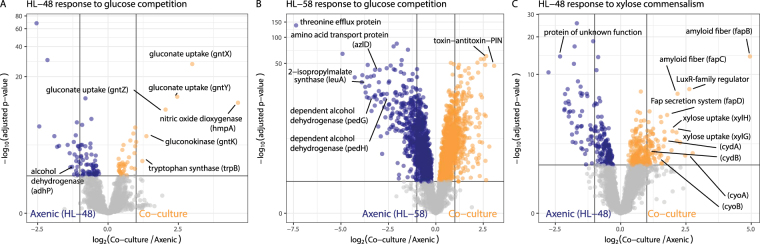
Volcano plots show the magnitude and significance of differentially expressed genes from each treatment of interspecies partnership. Thresholds are shown as solid lines indicating fold changes ≥ 2 and statistical significance defined by an adjusted p-value ≤ 0.05. Genes described in the main text are labelled. (**A** and **B**) Transcriptional responses of *Halomonas* HL-48 and *Marinobacter* HL-58 to competition for glucose. (**C**) Transcriptional responses of *Halomonas* HL-48 non-competitive/commensal cultivation with *Marinobacter* HL-58 on xylose. Note that differential gene expression from *Marinobacter* HL-58 in response to co-cultivation on xylose was not possible because *Marinobacter* HL-58 cannot be grown axenic on xylose.

Overall, *Halomonas* HL-48 showed a relatively limited transcriptional response to glucose supported co-cultivation (only 28 differentially expressed genes) as compared its competitor, *Marinobacter* HL-58. This observation is in accordance with the small change in growth rate observed from *Halomonas* HL-48 (only 6%). The specific genes upregulated by *Halomonas* HL-48 in response to glucose competition with *Marinobacter* HL-58 included a gluconokinase (*gntK*), a tripartite ATP-independent (TRAP-type) gluconate uptake system (*gntXYZ*), a tryptophan synthase (*trpB*) and a nitric oxide dioxygenase (*hmpA*) (Fig. 3A). The other 22 differentially expressed genes from *Halomonas* HL-48 were downregulated in response to competitive co-cultivation. Of note was an alcohol dehydrogenase (*adhP*). Decreased expression of this gene likely plays a role modulating extracellular ethanol concentration. The co-culture contained less ethanol than the axenic *Halomonas* HL-48 culture (47.6% decrease from axenic control; p < 0.001; pairwise t-test with Holm correction) (Fig. 1E).

*Marinobacter* HL-58 showed broad transcriptional responses to competitive co-cultivation (Fig. 3B). Competition for glucose induced upregulation of 257 genes. The highest differential expression (up to 8-fold change) was observed in stress response genes encoding for PilT N-terminal (PIN) domain proteins. This family has been shown to exhibit RNase activity (as part of the type II toxin-antitoxin system), and have been implicated in the attenuation of protein synthesis and retardation of cell growth under stress conditions^16,17^. *Marinobacter* HL-58 downregulated 255 genes in response to competitive co-cultivation. Of these, genes involved with the transport and metabolism of amino acids showed the largest fold changes (up to 140-fold) including a 2-isopropylmalate synthase (*leuA*), a branched chain amino acid transporter (*azlD; CD01DRAFT_3656*) and a putative threonine efflux protein (*CD01DRAFT_1793*). Genes encoding for an alcohol dehydrogenase and uptake system (*pedGH*) were also down regulated corresponding to a 23% decrease in mean ethanol concentration (p = 0.101; pairwise t-test with Holm correction) observed in co-culture as compared to the *Marinobacter HL-58* axenic control. The relatively large number of differentially regulated genes are an indication that competition with *Halomonas* HL-48 induced a strong phenotypic shift in *Marinobacter* HL-58.

In commensal co-culture, *Halomonas* HL-48 genes associated with transport, regulation, central carbon metabolism and energy metabolism showed a stronger differential in response to partnership than was observed in competitive co-culture. Under these growth conditions, *Halomonas* HL-48 maintained exclusive metabolic access to the only exogenous carbon and electron source, xylose. We identified 147 differentially expressed *Halomonas* HL-48 genes (Fig. 3C). Of these, 63 genes were upregulated, including an ABC-type xylose uptake system (*xylGH*) and terminal oxidase subunits (*cydAB* and *cyoAB*). The genes observed to be the most upregulated during commensal co-cultivation (up to 19.5-fold change) encode for amyloid fiber nucleation and secretion (*fapBCDE*) and LuxR-family transcriptional regulator (*CY41DRAFT_2487*). The remaining 84 differentially expressed *Halomonas* HL-48 genes were downregulated. Many of these were classified as hypothetical or unknown proteins including those showing the strongest downregulation (up to 6-fold change). This result supports the concept that microbial species exert either direct or indirect regulation over each other’s transcriptional processes and, therefore, behavior. The growth and molecular phenotypes of each species was context-specific and dependent on both the nutrient availability and interaction type of the co-culture.

## Discussion

Traditionally, the context under which microbial populations interact positively or negatively has been described by evaluating the growth phenotypes of cells within different environments. Specifically, the outcome from a competition experiment has almost exclusively been described by simple growth kinetics, e.g., by parameterizing Monod-type growth kinetics^18,19^ and comparing maximum specific growth rates and the affinities that different species have for substrates. We have gone beyond this classical method of investigation and examined functions that are responsive to either competitive or commensal conditions by examining the dynamics of the molecular phenotypes of each species. We learned that the relationships between interacting bacteria – competitive versus commensal – has a significant impact on their metabolic activity and growth rate. The results of this study clearly demonstrated that distinct growth and molecular phenotypes emerge from interspecies partnerships that can be induced via co-cultivation and that different nutrient conditions dictate how these partnerships will unfold.

Under competition, when glucose was the sole carbon source, the *Halomonas* HL-48 was able to outcompete *Marinobacter* HL-58 with very little change in its transcriptional profile or specific growth rate. *Halomonas* HL-48 was essentially unchanged from competitive partnership with *Marinobacter* HL-58. To arrive at this conclusion we compared the number of differentially expressed *Halomonas* HL-48 genes from the glucose, co-cultivation treatment (only 28 in total) to the other treatments derived from this study (≥ 147 differentially expressed genes). In sharp contrast, *Marinobacter* HL-58 was highly sensitive to competition for glucose; it showed strong shifts in both growth and molecular phenotypes. The competitive relationship was essentially one-sided in that *Halomonas* HL-48 gene expression was virtually unaffected, while *Marinobacter* HL-58 showed broad changes. This result indicates that the molecular phenotype that *Halomonas* HL-48 exhibits under glucose minimal media is robust against competition with a slower growting species.

Conversely, co-culturing under xylose showed that both *Halomonas* HL-48 and *Marinobacter* HL-58 exhibited substantial phenotypic changes (relative to axenic culture). This indicates that a two-way relationship had been established: *Marinobacter* HL-58 was consuming metabolites produced by *Halomonas* HL-48, and likely *Halomans* HL-48 was also responding to signals or metabolites produced (or removed) by *Marinobacter* HL-58. This result highlights a broader concept: that microbial species respond to one another as part of their environment and that they exert direct and/or indirect regulation over each other’s transcriptional and translational processes^11,14^. The specific phenotype of any population of species is directly influenced by the composition and metabolic functions of other species sharing the same the environment.

Competition for resources is a major determinant of ecosystem-level function. Many experiments show how different species, or variants of the same species, modulate relationships between growth rate and substrate availability in order to outcompete other populations^5^. Revealing the underlying biological principles that shape microbial community formation and function requires insight into the specific mechanisms of metabolic interaction. For instance, we observed a decrease in the specific growth rate of *Marinobacter* HL-58 in response to a competitive partnership. Yet only by examining genome-resolved transcriptomic data were we able to gain specific mechanistic insight into this response, for instance the upregulation of a type II toxin-antitoxin system (Fig. 3B). Assuming that increased transcript abundances result in increased RNase-like protein expression, this mechanism may have retarded cell growth in response to competition with *Halomonas* HL-48. Thus, our results go beyond simple observation of competition and commensalism; they provide a starting place to understand how genome-encoded functions dictate the outcomes of interspecies partnerships under specific environmental contexts.

A fundamental question in microbial ecology is whether species will co-exist in a given environment. Model microbial communities and co-cultures are useful tools for interrogating competitive and commensal relationships between species that might lead to competitive exclusion or co-existence. A prime example was presented by Christensen *et al*. in a study that evaluated the interactions between a *Pseudomonas putida* and an *Acinetobacter* species in well-mixed cultivation, as compared to biofilm growth^20^. They found that well mixed (i.e., chemostat) growth with limiting concentrations of benzyl alcohol resulted in strong competition where one species (*Acinetobacter*) outcompeted strongly the other. However, when given the same substrate under a different environmental context, the same two species exhibited commensal interactions that resulted in a biofilm formation mediated by co-existence and in the exchange of a byproduct metabolite (benzoate).

Another classical example of community-level phenotype switching – from competitive to commensal – has been observed and modeled in phytoplankton-bacteria interactions^21,22^. In this case, when bacteria and algae (previously exemplified using the diatom species, *Skeletonema costatum*) are limited by the same mineral nutrients (i.e., phosphorus), bacteria often show greater uptake affinities. The algae, however, respond to this competitive partnership by producing more extracellular organic carbon (compared to axenic, substrate limited growth) in order to stimulate their bacterial community members. This paradoxical state has been described as a “competitive-commensal” behavior that results in stable co-existence of both trophic types. Our current, genome-informed study significantly expands upon these classical reports by providing greater detail into the observation that microorganisms change their behavior based on interspecies partnerships and on the environmental context under which these partnerships occur.

## Conclusions

This study was driven by two fundamental questions: 1) *how do the phenotypes of two naturally co-existing species respond to partnership as compared to axenic growth?* and 2) *how do growth and molecular phenotypes of these species change with respect to competitive and commensal interactions?* Each species responded to partnership by changing both its growth and molecular phenotype from what could be observed during axenic growth under the same conditions. During competitive co-cultivation, the dominant species (*Halomonas* HL-48) responded robustly to the effects of partnership and maintained essentially the same molecular phenotype as assayed by global transcriptomics. *Marinobacter* HL-58, was the loser of the glucose competition and showed high sensitivity to partnership observed in the growth and molecular phenotypes. However, when this same binary partnership was induced in the presence of xylose, competition ceased and the co-culture obtained a commensal phenotype. *Halomonas* HL-48 – the xylose consumer – exhibited broad transcriptional response to partnership even though its growth phenotype remained similar. This naturally existing model system provides a basic framework for advancing our overall mechanistic understanding of how different environmental conditions prompt the expression of genome-encoded functions and therefore dictate the outcomes of interspecies partnerships.

## Methods

### Bacterial strains and cultivation

Two heterotrophic isolates were used for this study: *Halomonas* sp. HL-48 and *Marinobacter* sp. HL-58. These strains were co-isolated from Hot Lake, a MgSO_4_-dominated hypersaline ecosystem in North-Central Washington state^6^. These strains were grown under both axenic and co-culture conditions in a defined media named Hot Lake Heterotrophic (HLH) medium: 400 mM MgSO_4_·7H_2_O, 80 mM Na_2_SO_4_, 20 mM KCl, 0.189 mM Na_2_CO_3_, 0.175 mM K_2_HPO_4_, 5 mM NH_4_Cl, 10 mM TES, 22.3 µM NH_4_Fe(III) citrate, 10 mM NaHCO_3_. HLH was amended with 1× Wolfe’s vitamins^23^ and with either glucose or xylose (to 5 mM) as the sole carbon source. Basic growth/no-growth experiments of *Marinobacter* HL-58 were performed on the same HLH medium contain 5 mM of either sodium acetate or ethanol as the sole carbons source. Cultivation was performed in 150 ml, non-baffled Erlenmeyer flasks agitated at 150 rpm and held at 30 °C and buffered to pH 8. Inoculum for each treatment was derived from axenic starter cultures grown in HLH + 5 mM glucose and harvested during mid-log growth phase. The cells used as inoculum were collected by centrifugation, rinsed twice with the respective media for a given treatment (plus or minus glucose or xylose, respectively) and re-suspended to a starting optical density (OD600) of 0.01. Inoculation of each species was performed in equal ratios which is consistent with previous observations of these species and representative taxa maintaining similar relative abundances in more complex consortia and within the natural Hot Lake ecosystem^6,24^. The time course batch growth experiments were performed in three biological replicates. End point batch growth experiments were repeated as five biological replicates to provide material for the metabolomics and transcriptomics described below.

### Fluorescence activated cell sorting and co-culture growth kinetics

For the purposes of enhancing the species-specific enumeration of cells in co-culture, *Marinobacter* sp. HL-58 was labeled with a green fluorescent protein (GFP) expressed from a pPCRPROM29–2 plasmid (Supplemental Figure S1). To enable co-cultivation under kanamycin selection (50 µg ml^−1^), *Halomonas HL-48* was transformed with a pBBR-MCS2 plasmid maintaining a kanamycin resistance gene^25^. Conjugal transfer for each plasmid-strain pair was performed using the *Escherichia coli WM3064* donor strain by a previously described method^26^. The relative abundance of each species in co-culture was measured from cell broth fixed in 4% paraformaldehyde and processed through a BD Influx Fluorescence Activated Cell Sorter (FACS, BD Biosciences, San Jose, CA). Using the 488 nm excitation from a Sapphire LP laser (Coherent Inc., Santa Clara, CA) at 100 mW, samples were analyzed using a 70 μm nozzle. Optimization and calibration of the FACS was performed before each analysis using 3 μm Ultra Rainbow Fluorescent Particles (Spherotech, Lake Forest, IL). The ratio of the two distinct populations of cells, GFP labeled and unlabeled, within a mixed microbial community were identified from 50,000 recorded cells using size and complexity gates with FCS Express (Los Angeles, CA) flow cytometry software. Species-specific growth curves were produced from co-cultures by multiplying the OD600 measurements (proportional total biomass) by relative abundance ratios, derived across log-phase sampling points. FACS cell counting was replicated by two-three biological replicates derived as subset samples from the time course batch growth experiments; FACS data is shown in Supplemental Figure S2.

### Microscopy

The samples were stained for 10 minutes with 10 μg/ml wheat germ agglutinin conjugated with Alexa Fluor 647 (WGA-647) (ThermoFisher, Waltham, MA). Samples were then washed with PBS and imaged. Images were acquired at 0.39 μm z-steps on a Zeiss LSM 710 scanning head confocal microscope equipped with an Airyscan detector (Carl Zeiss MicroImaging GmbH, Jena, Germany) with a Zeiss a Plan-Apochromat 100x/1.46 DIC M27 75 mm objective. Excitation lasers were 405 and 633 nm for the green and red emission channels, respectively. GFP was detected with a 495–550 nm filter and WGA-647 was detected with a 645 nm LP filter. Laser dwell times were 1.65 μs for both channels. Image analysis (2D and 3D) was conducted using Volocity (PerkinElmer, Waltham, MA).

### External metabolite detection

NMR analysis was used to quantitatively measure and compare extracellular metabolites in axenic and co-culture conditions. Cultures were sampled (1 ml) during mid-log growth phase, which corresponded to 20 and 33 h for each treatment (plus or minus glucose or xylose, respectively). Samples from each treatment were collected from 5 biological replicates; passed through 0.22 µm membranes (EMD Millipore Corporation, Billerica, MA); immediately flash frozen in liquid nitrogen and stored at −80 °C. Prior to NMR analysis, these samples were thawed and spiked with 1/10 volume of a solution containing 5 mM 2,2-dimethyl-2-silapentane-5-sulfonate (DSS) and 0.2% sodium azide (Sigma-Aldrich, St. Louis, MO) dissolved in 100% deuterium oxide (D_2_O, Cambridge Isotope Laboratories, Tewksbury, MA). NMR data was acquired on a Varian Direct Drive (VNMRS) 600 MHz spectrometer (Agilent Technologies, Santa Clara, CA) equipped with a Dell Precision T3500 Linux workstation running VNMRJ 4.2. The spectrometer system was outfitted with a Varian triple resonance salt-tolerant cold probe with a cold carbon preamplifier. A Varian standard one-dimensional proton nuclear Overhauser effect spectroscopy (NOESY) with presaturation (TNNOESY) was collected on each sample, using the following data collection protocol: nonselective 90 degree excitation pulse, a 100 millisecond mixing time, acquisition time of 4 s, a presaturation delay of 1.5 s, spectral width of 12 ppm, and temperature control set to 25 °C. Collected spectra were analyzed using Chenomx 8.2 software (Edmonton, Alberta, Canada), with quantifications based on spectral intensities relative to 0.5 mM DSS. Metabolite measurements were collected for five biological replicates.

### mRNA isolation and nucleic acid sequencing

Cells were collected by  centrifugation (7000 RPM, 4 min at 4 °C) from 50–100 ml of cell broth sampled from the mid-log phase (20 and 33 h) of each axenic and co-culture treatment (plus or minus glucose or xylose, respectively). The cell pellets were immediately flash frozen in liquid nitrogen and stored at −80 °C to await RNA isolation. Total RNA was extracted using TRIzol Reagent (Invitrogen, Carlsbad, CA), followed by genomic DNA removal and cleaning using RNase-Free DNase Set kit and the Mini RNeasy kit (Qiagen, Hilden, Germany). Ribosomal RNA was depleted from each sample using Ribo-Zero (bacteria) rRNA Removal Kit (Illumina, San Diego, CA). The quality and integrity of the RNA was assessed on an Agilent 2100 Bioanalyzer; only samples with integrity numbers between 8 and 10 were selected for further analysis^27^. Template cDNA was prepared using the Applied Biosystems SOLiD Total RNA-Seq Kit (Life Technologies, Carlsbad, CA) according to manufacturer’s protocol. Sequencing was carried out using the SOLiD 5500XL protocol (Life Technologies Carlsbad, CA). The 50-base sequence reads were mapped to the genomes of *Halomonas* HL-48 and *Marinobacter* HL-58 using SOLiD LifeScope *v*. 2.5 software. The complete reference genomes have previously been published^7^. Nucleotide sequence is available through the European Nucleotide Archive (http://www.ebi.ac.uk/ena) under accessions GCA_000686925.1 (*Halomonas* HL-48) and GCA_000686085.1 (*Marinobacter* HL-58). The genomes were annotated integrating information from the following pipelines: 1) the DOE-JGI Microbial Genome Annotation Pipeline^28^, BlastKOALA^29^, and the RAST server^30^, as previously described^9^. Annotations are available on GitHub (see below) and through IMG (http://img.jgi.doe.gov). The total number of protein encoding genes identified for *Halomonas* HL-48 and *Marinobacter* HL-58 were 3381 and 3875, respectively. RNA-seq was performed on 3–5 biological replicates. After alignment raw counts were normalized and differentially expressed genes were identified using the R package DESeq2^31^. Differentially expressed genes are defined as those showing > 2-fold change in expression when comparing two conditions with an adjusted p-value of ≤0.05.

### Data repositories and reproducibility

RNA-seq data is available on The Gene Expression Omnibus (GEO; NCBI) under accession GSE103773. Minimally processed RNA-seq data along with the metadata for growth and metabolite analysis is also included along an R Markdown file that archives this study’s data analysis and graphing procedures GitHub: https://github.com/pnnl/khan-2017-co-culture-phenotypes.

## Electronic supplementary material

Supplementary Information

Supplementary data and results
