# Supplementary material for: Phenotypic responses to interspecies competition and commensalism in a naturally-derived microbial co-culture
Source: Sci Rep. 2018 Jan 10;8:297. doi: 10.1038/s41598-017-18630-1 (PMC5762899; doi:10.1038/s41598-017-18630-1)
Supplement: Supplementary file 1 — Supplementary Information [file 41598_2017_18630_MOESM1_ESM.doc]

# Phenotypic responses to interspecies competition and commensalism in a naturally-derived microbial co-culture

Nymul Khan^1^, Yukari Maezato^1^, Ryan S. McClure^1^, Colin J. Brislawn^1^, Jennifer M. Mobberley^1^, Nancy Isern^2^, William B. Chrisler^1,2^, Lye Meng Markillie^2^, Brett M. Barney^3^, Hyun-Seob Song^1^, William C. Nelson^1^, Hans C. Bernstein^1,4^*

^1^Biological Sciences Division, Pacific Northwest National Laboratory, Richland, WA, USA; ^2^ Environmental Molecular Sciences Laboratory, Pacific Northwest National Laboratory, Richland, Washington, USA; ^3^Department of Bioproducts and Biosystems Engineering, University of Minnesota, St. Paul, MN 55108; ^4^The Gene and Linda Voiland School of Chemical Engineering and Bioengineering, Washington State University, Pullman, WA, USA

**** Correspondence:***Hans C. Bernstein, Biological Sciences Division, Pacific Northwest National Laboratory, P.O. Box 999, MS-IN: J4-18, Richland, WA 99352 ([Hans.Bernstein@pnnl.gov](mailto:Hans.Bernstein@pnnl.gov))

## Supplementary Methods for Data Analysis

#### About this document

This is an R Markdown document. Markdown is a simple formatting syntax for authoring HTML, PDF, and MS Word documents. For more details on using R Markdown see [http://rmarkdown.rstudio.com](http://rmarkdown.rstudio.com/).

This project makes use of many packages, especially: DESeq2 from Bioconductor <http://bioconductor.org/packages/release/bioc/html/DESeq2.html>.

The goal of this document it to provide a reproducible comprehensive overview of data analysis methods

## Library Setup:

library("checkpoint") #Part of MS R Open to make this software stack more reproducible
library("knitr") #package for report generation; assists with R markdown formatting
checkpoint("2017-07-01", use.knitr = T)

library("dplyr") #package for manipulating data frames
library("ggplot2") #plotting package
library("reshape2") #package for manipulating data frames
library("ggrepel") #add on to ggplot2 for generating labels
library("cowplot") #add on to ggplot2 for building "publication ready plots"
library("viridis") #color palette package
library("broom") #tidy stats outputs
library("kableExtra")

library("DESeq2") #significance testing for RNA-seq data

knitr::opts_chunk$set(cache=TRUE)
theme_set(theme_bw())
set.seed('711')

# DESeq is installed through bioconductor
source("https://bioconductor.org/biocLite.R")
biocLite("DESeq2", suppressUpdates = T)

## Import data:

options(stringsAsFactors = FALSE)

#info of treatments and replicates
info.48 <- read.csv("../data/Condition_Info_HL_48.csv")
info.58 <- read.csv("../data/Condition_Info_HL_58.csv")

#growth curves and GFP-enabled FACS data
grow <- read.csv("../data/Growth.2.csv")

#gene annotation files
an.48 <- read.csv("../data/HL_48_Neat.csv")
an.58 <- read.csv("../data/HL_58_Neat.csv")

#raw gene rollup counts
raw.48 <- read.csv("../data/HL48_Raw_Counts.csv")
raw.58 <- read.csv("../data/HL58_Raw_Counts.csv")

meta <- read.csv("../data/Metabolites.2.csv")

## Process species-specific growth kinetics

Bulk growth curves measured for each axenic and co-culture treatment the relative abundance of each species in co-culture is reported as the fraction of cells maintaining GFP these fractions were measured via FACS

#GFP.num <- data.frame(grow$OD600*grow$HL58.GFP.frac)
#colnames(GFP.num) <- "HL-58"
#parent.num <- data.frame(grow$OD600*grow$HL48.frac)
#colnames(parent.num) <- "HL-48"
#grow <- cbind(grow, GFP.num, parent.num)
#vars <- c("HL.58.GFP.frac", "parent.num") #try to pull frac values out of data frame
#grow <- cbind(grow[!vars], GFP.num, parent.num) #try to add abs values
t1 <- melt(grow, id.vars = c("Treatment", "Cult", "Species", "Substrate",
 "Sample.ID", "Replicate", "Time.h", "GFP.frac", "parent.frac"))
#long format data

p.gro <- ggplot(t1, aes(x = Time.h, y = value, color = variable)) +
 geom_point() +
 #scale_y_log10() +
 facet_grid(Cult ~ Substrate, scales = "free_x") +
 theme(strip.background = element_blank(), legend.position = "none") +
 geom_smooth(se = F)
p.gro

## `geom_smooth()` using method = 'loess'


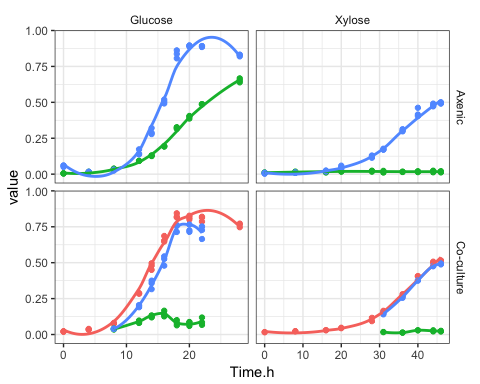


t1 %>% dim
t2 <- subset(t1, Time.h > 0)

t2.g <- subset(t2, Substrate=="Glucose" & Time.h < 18)
t2.x <- subset(t2, Substrate=="Xylose" & Time.h < 45)
t2 <- rbind(t2.g, t2.x)

t2 %>% dim

# Let's see if we can add slopes and p-values to these graphs
# See https://stackoverflow.com/questions/17022553/adding-r2-on-graph-with-facets
# Also used on https://github.com/pnnl/bernstein-2017-productivity-and-diversity-2/

df <- subset(t2, Substrate=="Xylose" & Cult=="Axenic" & variable == "HL.48")
df <- subset(t2, Substrate=="Xylose" & Cult=="Axenic" & variable == "Total.OD600")
head(df)
dim(df)

lm_eqn_growth = function(df){

 if(all(is.na(df$value))) return("")
 # Super important! Return an empty string for the missing values.

 m = summary(lm(log(value) ~ Time.h, df)) # Hardcoded to my data
 m

 m$coefficients
 m$coefficients[2] # Slope of Time.h
 m$coefficients[8] # P value of Time.h

 if(m$coefficients[8] < 0.001) {
 outputp <- "0.001"
 }else{
 outputp <- round(m$coefficients[8], digits = 3)
 }

 eq <- substitute(
 atop("slope ="~slope, "p-value ="~pr), # Two lines
 #"slope ="~slope~","~~R^2~"="~r2, # One line
 list(slope = signif(m$coefficients[2], digits = 3),
 pr = outputp)
 )
 return(as.character(as.expression(eq)))
}

lm_eqn_growth(df)

eqns <- by(t2, INDICES = list(t2$Substrate, t2$Cult, t2$variable), lm_eqn_growth)
eqns

df2 <-
 data.frame(eq = c(eqns),
 Substrate = rep(c("Glucose", "Xylose"), 6),
 Cult = rep(c("Axenic", "Axenic", "Co-culture","Co-culture"), 3),
 variable = c(rep("Total.OD600", 4), rep("HL.58", 4), rep("HL.48", 4))
 ,graphx = c(10, 10, 8, 20, 14, 35, 14, 22, 8, 20, 8, 20)
 ,graphy = c(.1, .1, .7, .7, .02, .05, .02, .014, .6, .6, .3, .3)
)

df2
df2 <- subset(df2, eq != "") # Remove the empty lines

p.gro <- ggplot(t2, aes(x = Time.h, y = value, color = variable)) +
 geom_point() +
 scale_y_log10() +
 geom_text(data = df2, aes(x = graphx, y = graphy, label = eq), parse = TRUE, show.legend = F) +
 facet_grid(Cult ~ Substrate, scales = "free_x") +
 labs(x = "Time (h)", y = expression(log(OD[600])), parse = T) +
 scale_color_manual(values = c("#999999", "#6DCD59FF", "#482878")) +
 theme(strip.background = element_blank(), legend.position = "none")
p.gro + geom_smooth(method = "lm", fill = "#CCCCCC", show.legend = F)


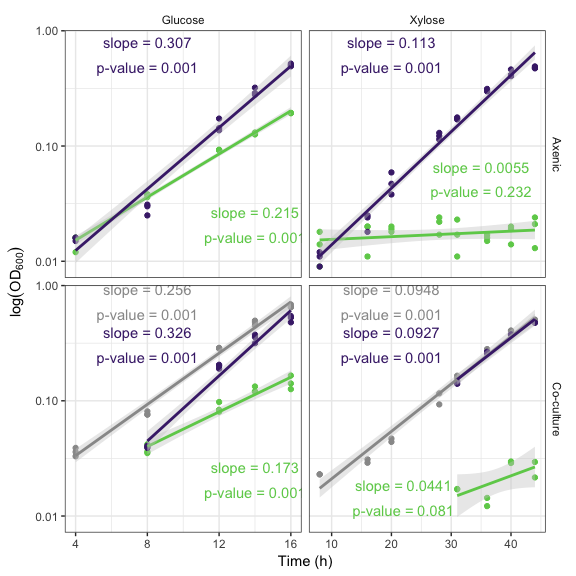


ggsave("figures/fig1-parts/fig1-growth.pdf", width = 120, units = "mm", height = 90, scale = 1.5)


#### Try it flipped!
# p.gro <- ggplot(t2, aes(x = Time.h, y = value, color = variable)) +
# geom_point() +
# scale_y_log10() +
# geom_text(data = df2, aes(x = graphx, y = graphy, label = eq), parse = TRUE, show.legend = F) +
# facet_grid(Substrate ~ Cult, scales = "free_x") +
# labs(x = "Time (h)", y = expression(log(OD[600])), parse = T) +
# scale_color_manual(values = c("#999999", "#6DCD59FF", "#482878")) +
# theme(strip.background = element_blank(), legend.position = "none")
# p.gro + geom_smooth(method = "lm", fill = "#CCCCCC", show.legend = F)
#
# # Note how "free_x" no longer applied because Time.h must be consistent
# while stacked.
# # Positions of labels also make less sense.
# ggsave("figures/fig1-parts/fig1-growth-flipped.pdf", width = 120, units = "mm", height = 90, scale = 1.5)

Cutaway of one frame from the above figure.

Confirm this goal: Show full time course of one block of the ANOVA design, without dropping timepoints or using a log10() transform.

# Start with the full t1 subset
t3 <- subset(t1, Substrate=="Glucose" & Cult == "Co-culture")

p.gro <- ggplot(t3, aes(x = Time.h, y = value, color = variable)) +
 geom_point() +
 facet_grid(Cult ~ Substrate, scales = "free_x") +
 labs(x = "Time (h)", y = expression(OD[600]), parse = T) +
 scale_color_manual(values = c("#999999", "#6DCD59FF", "#482878")) +
 theme(strip.background = element_blank(), legend.position = "none")
p.gro + geom_smooth(fill = "#CCCCCC", show.legend = F)

## `geom_smooth()` using method = 'loess'


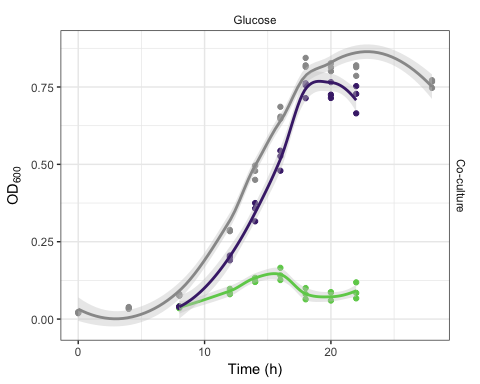


ggsave("figures/fig1-parts/fig1-growth-cutaway.pdf", width = 60, units = "mm", height = 50, scale = 1.5)

## `geom_smooth()` using method = 'loess'

# All data, for use as a sub figure.
# We add back in the legend, so this does not need the legend in another part of a larger figure.
p.gro <- ggplot(t1, aes(x = Time.h, y = value, color = variable)) +
 geom_point() +
 facet_grid(Cult ~ Substrate, scales = "free_x") +
 labs(x = "Time (h)", y = expression(OD[600]), parse = T) +
 scale_color_manual(values = c("#999999", "#6DCD59FF", "#482878"), name = "Treatment") +
 theme(strip.background = element_blank(), legend.position = c(.1,.88))
p.gro + geom_smooth(fill = "#CCCCCC", show.legend = F)

## `geom_smooth()` using method = 'loess'


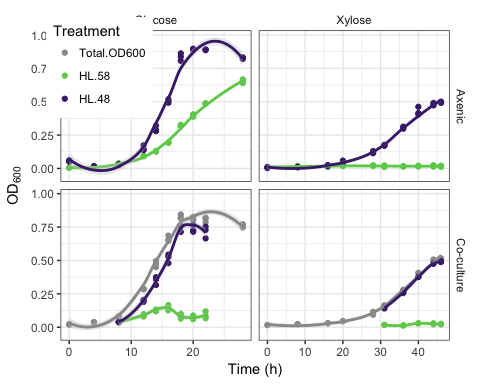


ggsave("figures/fig1-parts/fig1-growth-cutaway-full.pdf", width = 120, units = "mm", height = 90, scale = 1.5)

## `geom_smooth()` using method = 'loess'

## Process external metabolites

NMR metabolomics data derived from culture filtrate

Analyze and compare the concentrations of external metabolites across treatments.

summary(meta)
str(meta)

met <- filter(meta, Metabolite != "DSS-d6 (Chemical Shape Indicator)")
met <- filter(met, Experiment.Date != "Control March 2017")
met <- filter(met, Metabolite != "Glucose")
met <- filter(met, Metabolite != "Xylose")
met$Microbe <- factor(met$Microbe, levels = c("HL-48", "HL-48/58", "HL-58"))

g.met <- ggplot(met, aes(Metabolite, Conc.uM, color = Microbe))
g.met +
 geom_boxplot(width = .5) +
 labs(y = expression(paste("Concentration (", mu, M, ")")), color = "Treatment: ", x = "") +
 facet_wrap(~Substrate, ncol = 2) +
 scale_color_manual(values = c("#482878", "#777777", "#6DCD59FF")) +
 theme(strip.background = element_blank(), axis.text.x = element_text(angle = -20, hjust=0)
 ,legend.position = "top", legend.direction = "horizontal"
 ,plot.margin = margin(0, 1, 0, .5, "lines")
 #,legend.position = "none", plot.margin = unit(c(0, 0, 0, .5), "lines")
 )

## Warning: Removed 3 rows containing non-finite values (stat_boxplot).


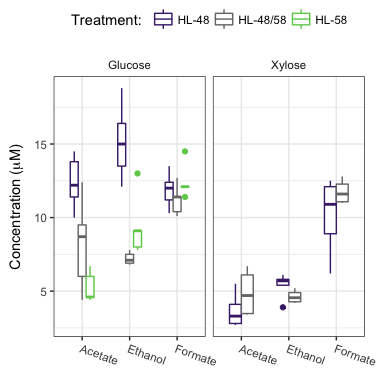


# wide; full width of growth curve
ggsave("figures/fig1-parts/fig1-mets-wide1.pdf", width = 120, units = "mm", height = 60, scale = 1.3)

## Warning: Removed 3 rows containing non-finite values (stat_boxplot).

# square; half width of growth curve
ggsave("figures/fig1-parts/fig1-mets.pdf", width = 60, units = "mm", height = 50, scale = 1.4)

## Warning: Removed 3 rows containing non-finite values (stat_boxplot).

# Try it flipped (matches flipped growth graph)
# g.met +
# geom_boxplot(width = .5) +
# labs(y = expression(paste("Concentration (", mu, M, ")")), color = "Treatment", x = "") +
# facet_wrap(~Substrate, ncol = 1, strip.position = "right") +
# scale_color_manual(values = c("#482878", "#777777", "#6DCD59FF")) +
# theme(strip.background = element_blank(), axis.text.x = element_text(angle = -20, hjust=0))
#
# ggsave("figures/fig1-parts/fig1-mets-flipped.pdf", width = 60, units = "mm", height = 50, scale = 1.4)

We need to do t-test to establish differences between ethanol and acetate abundances between treatments. This can be output as a supplementary table or kable in the markdown. You will see some notes in the Results text corresponding to this as well.

met %>% head

## Treatment Sample.Number Experiment.Date Replicate Metabolite Conc.uM X
## 1 HL-48 G N1 Main Jan 2017 1 Acetate 11.4 G
## 2 HL-48 G N1 Main Jan 2017 1 Ethanol 18.8 G
## 3 HL-48 G N1 Main Jan 2017 1 Formate 11.2 G
## 4 HL-48 G N2 Main Jan 2017 2 Acetate 10.0 G
## 5 HL-48 G N2 Main Jan 2017 2 Ethanol 15.0 G
## 6 HL-48 G N2 Main Jan 2017 2 Formate 12.4 G
## Substrate Cult Microbe
## 1 Glucose Axenic HL-48
## 2 Glucose Axenic HL-48
## 3 Glucose Axenic HL-48
## 4 Glucose Axenic HL-48
## 5 Glucose Axenic HL-48
## 6 Glucose Axenic HL-48

# Thanks to the magic of dplyr, broom, and default stats, we can do this:
met %>% group_by(Substrate, Metabolite) %>%
 do(tidy(pairwise.t.test(.$Conc.uM, .$Microbe, p.adj = "holm"))) %>%
 kable() %>% kable_styling(full_width = F)

## Currently generic markdown table using pandoc is not supported.

| Substrate | Metabolite | group1 | group2 | p.value |
| --- | --- | --- | --- | --- |
| Glucose | Acetate | HL-48/58 | HL-48 | 0.0200808 |
| Glucose | Acetate | HL-58 | HL-48 | 0.0006665 |
| Glucose | Acetate | HL-58 | HL-48/58 | 0.0529371 |
| Glucose | Ethanol | HL-48/58 | HL-48 | 0.0000932 |
| Glucose | Ethanol | HL-58 | HL-48 | 0.0010511 |
| Glucose | Ethanol | HL-58 | HL-48/58 | 0.1014061 |
| Glucose | Formate | HL-48/58 | HL-48 | 0.7321162 |
| Glucose | Formate | HL-58 | HL-48 | 0.7321162 |
| Glucose | Formate | HL-58 | HL-48/58 | 0.3372185 |
| Xylose | Acetate | HL-48/58 | HL-48 | 0.2453340 |
| Xylose | Ethanol | HL-48/58 | HL-48 | 0.1623205 |
| Xylose | Formate | HL-48/58 | HL-48 | 0.2733803 |

# Note we are using the 'holm' correction because it's 'uniformly more powerful' then 'bonf'.
# If we want to switch to Bonferroni because it's popular or we want CIs, that's ok too.

# let's pull mean values too
#met %>% group_by(Substrate, Metabolite, Microbe) %>% summarise(mean.Conc.uM = mean(Conc.uM, na.rm = T))
#met %>% group_by(Substrate, Metabolite, Microbe) %>% summarise(med.Conc.uM = median(Conc.uM, na.rm = T))

met %>% group_by(Substrate, Metabolite, Microbe) %>% summarise(med.Conc.uM = median(Conc.uM, na.rm = T)) %>%
 filter(Substrate == "Xylose", Metabolite == "Ethanol")

## # A tibble: 2 x 4
## # Groups: Substrate, Metabolite [1]
## Substrate Metabolite Microbe med.Conc.uM
## <chr> <chr> <fctr> <dbl>
## 1 Xylose Ethanol HL-48 5.70
## 2 Xylose Ethanol HL-48/58 4.55

5.7-4.55

## [1] 1.15

(5.7-4.55)/5.7

## [1] 0.2017544

met %>% group_by(Substrate, Metabolite, Microbe) %>% summarise(mean.Conc.uM = mean(Conc.uM, na.rm = T)) %>%
 filter(Substrate == "Xylose", Metabolite == "Ethanol")

## # A tibble: 2 x 4
## # Groups: Substrate, Metabolite [1]
## Substrate Metabolite Microbe mean.Conc.uM
## <chr> <chr> <fctr> <dbl>
## 1 Xylose Ethanol HL-48 5.380
## 2 Xylose Ethanol HL-48/58 4.625

5.380-4.625

## [1] 0.755

(5.380-4.625)/5.380

## [1] 0.1403346

# Line 156
met %>% group_by(Substrate, Metabolite, Microbe) %>% summarise(mean.Conc.uM = mean(Conc.uM, na.rm = T)) %>%
 filter(Substrate == "Glucose", Metabolite == "Ethanol")

## # A tibble: 3 x 4
## # Groups: Substrate, Metabolite [1]
## Substrate Metabolite Microbe mean.Conc.uM
## <chr> <chr> <fctr> <dbl>
## 1 Glucose Ethanol HL-48 15.16
## 2 Glucose Ethanol HL-48/58 7.22
## 3 Glucose Ethanol HL-58 9.40

15.16/7.22 # subtract 1 to get 'percent increase'

## [1] 2.099723

# line 174
met %>% group_by(Substrate, Metabolite, Microbe) %>% summarise(mean.Conc.uM = mean(Conc.uM, na.rm = T)) %>%
 filter(Substrate == "Glucose", Metabolite == "Ethanol")

## # A tibble: 3 x 4
## # Groups: Substrate, Metabolite [1]
## Substrate Metabolite Microbe mean.Conc.uM
## <chr> <chr> <fctr> <dbl>
## 1 Glucose Ethanol HL-48 15.16
## 2 Glucose Ethanol HL-48/58 7.22
## 3 Glucose Ethanol HL-58 9.40

(7.22/9.40) # relative

## [1] 0.7680851

(7.22- 9.40) / 9.4 # subtract 1 to get 'percent increase'

## [1] -0.2319149

## Process RNAseq data

Volcano plots!

#### Normalize RNAseq data to RPKM

RNA seq data processing for Halomonas HL-48; generate normalized counts in RPKM

dfcountData <- data.frame(raw.48, row.names = 1)
dfcolData <- data.frame(info.48, row.names = 1)

dds <- DESeqDataSetFromMatrix(countData = dfcountData, colData = dfcolData, design = ~condition)

## Warning in DESeqDataSet(se, design = design, ignoreRank): some variables in
## design formula are characters, converting to factors

dds.48 <- DESeq(dds)

## estimating size factors

## estimating dispersions

## gene-wise dispersion estimates

## mean-dispersion relationship

## -- note: fitType='parametric', but the dispersion trend was not well captured by the
## function: y = a/x + b, and a local regression fit was automatically substituted.
## specify fitType='local' or 'mean' to avoid this message next time.

## final dispersion estimates

## fitting model and testing

notAllZero <- (rowSums(counts(dds.48)) > 0)
vsd <- varianceStabilizingTransformation(dds.48)

## -- note: fitType='parametric', but the dispersion trend was not well captured by the
## function: y = a/x + b, and a local regression fit was automatically substituted.
## specify fitType='local' or 'mean' to avoid this message next time.

HL48.norm.count <- data.frame(assay(vsd[notAllZero,]))
HL48.norm.count <- add_rownames(HL48.norm.count, "GeneID")

## Warning: Deprecated, use tibble::rownames_to_column() instead.

HL48.norm.count <- merge(an.48, HL48.norm.count, by = "GeneID")

write.table(HL48.norm.count, file="RNA_seq_outputs/HL48_Norm_Exp_Values_annotated.csv", quote=FALSE, sep=",", row.names=FALSE, col.names=TRUE)
kable(head(HL48.norm.count))

| GeneID | Acc_num | Main_Role | Subrole | Product | Gene | T1_HL48G | T2_HL48G | T4_HL48G | T5_HL48G | T11_HL48G | T12_HL48G | T13_HL48G | T14_HL48G | T15_HL48G | T16_HL48X | T17_HL48X | T18_HL48X | T19_HL48X | T21_HL48X | T22_HL48X | T23_HL48X | T24_HL48X |
| --- | --- | --- | --- | --- | --- | --- | --- | --- | --- | --- | --- | --- | --- | --- | --- | --- | --- | --- | --- | --- | --- | --- |
| hotlake_ucc_124538 | CY41DRAFT_0125 | Nucleic acid metabolism | DNA replication_ recombination_ and repair | DNA repair protein radc |  | -5.7848388 | -6.3143024 | -4.4814389 | -4.5377806 | -4.5265101 | -4.8708085 | -5.510563 | -4.6542463 | -5.3449012 | -6.0842219 | -3.9634773 | -6.5836009 | -5.8550017 | -5.5382288 | -5.2826004 | -5.4470966 | -5.0667479 |
| hotlake_ucc_124539 | CY41DRAFT_0132 | Nucleic acid metabolism | DNA replication_ recombination_ and repair | Nucleotidyltransferase/DNA polymerase involved in DNA repair |  | 4.9963436 | 4.7277389 | 4.7600992 | 6.3692662 | 7.2207782 | 5.0379955 | 6.861618 | 6.0918492 | 5.2610346 | 9.1327328 | 9.3193559 | 9.1138284 | 9.3900502 | 9.1654333 | 9.0195327 | 9.1943398 | 4.6304036 |
| hotlake_ucc_124540 | CY41DRAFT_0143 | Mobile and extrachromosomal element functions | Selfish genetic elements | IS3 family transposase |  | 1.8254382 | 1.9322351 | 0.8025498 | 3.1816689 | 3.8016526 | 3.3107449 | 3.334614 | 3.0542134 | 1.7957520 | 0.5970179 | -0.2134157 | 0.0705615 | 0.0969528 | 0.9398863 | 0.6832929 | 0.9688571 | 2.4263460 |
| hotlake_ucc_124541 | CY41DRAFT_0145 |  |  | transcriptional regulator-like protein |  | 0.0961810 | 0.0142558 | -1.6310959 | 0.9014977 | -0.7069261 | 0.3712687 | 1.719177 | 0.2093218 | -0.2096073 | 1.5702723 | 0.9160743 | 1.9851819 | 0.7020047 | 2.3341311 | 1.7835076 | 1.1339884 | 0.6228251 |
| hotlake_ucc_124542 | CY41DRAFT_0180 |  |  | hypothetical protein |  | 0.0081833 | -0.1290377 | -0.5916545 | -1.1555539 | -1.8462097 | 0.5030828 | -1.057093 | -2.0216193 | 1.2537645 | -2.0979835 | -1.3978755 | -1.2436046 | -3.7383752 | -1.7227944 | -1.8882386 | -1.7831942 | 1.7534734 |
| hotlake_ucc_124543 | CY41DRAFT_0217 |  |  | transposase |  | -3.2836200 | -2.3520823 | -0.8251749 | -2.6913921 | -1.0130258 | -2.0514015 | -2.805127 | 0.4255340 | -1.2107125 | -3.6241258 | -4.7173176 | -2.2823046 | -2.8908062 | -1.9026651 | -4.4514642 | -3.1411675 | -2.9460297 |

# HL48.norm.count %>% head
# # So this simple df includes gene annotations and is normalized...
# dds.48 %>% assay() %>% head
# # ...while this is the full object, without normalization.
colData(dds.48)$condition %>% table

## .
## HL_48_58_G HL_48_58_X HL_48_G HL_48_X
## 5 4 4 4

General function for DESeq contrasts.

deseq_diff <- function(df, contrasts, annotations){
 # perform deseq contrast
 r <- results(df, contrasts)

 # Convert to table and relablel row names to GeneID
 r <- add_rownames(data.frame(r), "GeneID")

 # merge in annotations from file
 r.an <- merge(annotations, r, by = "GeneID")

 return(r.an)
}

generate differential expression output for HL-48 glucose competition treatment HL-48 glucose axenic vs. HL-58/HL-48 glucose co-culture

HL48.diff.comp <- deseq_diff(dds.48, c("condition","HL_48_58_G","HL_48_G"), annotations = an.48)

write.table(HL48.diff.comp, file="RNA_seq_outputs/HL48.diff.comp.csv", quote=FALSE, sep=",", row.names=FALSE, col.names=TRUE)
kable(head(HL48.diff.comp))

| GeneID | Acc_num | Main_Role | Subrole | Product | Gene | baseMean | log2FoldChange | lfcSE | stat | pvalue | padj |
| --- | --- | --- | --- | --- | --- | --- | --- | --- | --- | --- | --- |
| hotlake_ucc_124538 | CY41DRAFT_0125 | Nucleic acid metabolism | DNA replication_ recombination_ and repair | DNA repair protein radc |  | 9.761608 | 0.2435938 | 0.4007812 | 0.6077975 | 0.5433218 | 0.8554307 |
| hotlake_ucc_124539 | CY41DRAFT_0132 | Nucleic acid metabolism | DNA replication_ recombination_ and repair | Nucleotidyltransferase/DNA polymerase involved in DNA repair |  | 800.375384 | 0.3960024 | 0.3193219 | 1.2401357 | 0.2149252 | 0.6491502 |
| hotlake_ucc_124540 | CY41DRAFT_0143 | Mobile and extrachromosomal element functions | Selfish genetic elements | IS3 family transposase |  | 138.723430 | 0.4380131 | 0.2171244 | 2.0173366 | 0.0436604 | 0.3363009 |
| hotlake_ucc_124541 | CY41DRAFT_0145 |  |  | transcriptional regulator-like protein |  | 99.795097 | 0.1635539 | 0.2409007 | 0.6789266 | 0.4971844 | 0.8371530 |
| hotlake_ucc_124542 | CY41DRAFT_0180 |  |  | hypothetical protein |  | 60.185987 | 0.0298161 | 0.3886594 | 0.0767153 | 0.9388500 | 0.9854406 |
| hotlake_ucc_124543 | CY41DRAFT_0217 |  |  | transposase |  | 36.230425 | 0.5619926 | 0.3528957 | 1.5925176 | 0.1112685 | 0.5081949 |

generate differential expression output for HL-48 xylose commensalism treatment HL-48 xylose axenic vs. HL-58/HL-48 xylose co-culture

HL48.diff.cmns <- deseq_diff(dds.48, c("condition","HL_48_58_X","HL_48_X"), an.48)

write.table(HL48.diff.cmns, file="RNA_seq_outputs/HL48.diff.cmns.csv", quote=FALSE, sep=",", row.names=FALSE, col.names=TRUE)
kable(head(HL48.diff.cmns))

| GeneID | Acc_num | Main_Role | Subrole | Product | Gene | baseMean | log2FoldChange | lfcSE | stat | pvalue | padj |
| --- | --- | --- | --- | --- | --- | --- | --- | --- | --- | --- | --- |
| hotlake_ucc_124538 | CY41DRAFT_0125 | Nucleic acid metabolism | DNA replication_ recombination_ and repair | DNA repair protein radc |  | 9.761608 | 0.0039493 | 0.4815792 | 0.0082007 | 0.9934569 | 0.9963762 |
| hotlake_ucc_124539 | CY41DRAFT_0132 | Nucleic acid metabolism | DNA replication_ recombination_ and repair | Nucleotidyltransferase/DNA polymerase involved in DNA repair |  | 800.375384 | -0.3664611 | 0.3343952 | -1.0958924 | 0.2731259 | 0.5124676 |
| hotlake_ucc_124540 | CY41DRAFT_0143 | Mobile and extrachromosomal element functions | Selfish genetic elements | IS3 family transposase |  | 138.723430 | 0.5323011 | 0.2499474 | 2.1296527 | 0.0332003 | 0.1327357 |
| hotlake_ucc_124541 | CY41DRAFT_0145 |  |  | transcriptional regulator-like protein |  | 99.795097 | 0.1050630 | 0.2529892 | 0.4152866 | 0.6779321 | 0.8371175 |
| hotlake_ucc_124542 | CY41DRAFT_0180 |  |  | hypothetical protein |  | 60.185987 | 0.7602655 | 0.4311079 | 1.7635158 | 0.0778135 | 0.2392591 |
| hotlake_ucc_124543 | CY41DRAFT_0217 |  |  | transposase |  | 36.230425 | 0.2275172 | 0.4146091 | 0.5487511 | 0.5831763 | 0.7759768 |

generate differential expression output for HL-48 competition over commensalism HL-58/HL-48 xylose co-culture (cmns) vs. HL-58/HL-48 glucose co-culture (comp)

# New plot comparing treatments
HL48.diff.coculture <- deseq_diff(dds.48, c("condition","HL_48_58_G", "HL_48_58_X"), an.48)

write.table(HL48.diff.coculture, file="RNA_seq_outputs/HL48.diff.coculture.csv", quote=FALSE, sep=",", row.names=FALSE, col.names=TRUE)
kable(head(HL48.diff.coculture))

| GeneID | Acc_num | Main_Role | Subrole | Product | Gene | baseMean | log2FoldChange | lfcSE | stat | pvalue | padj |
| --- | --- | --- | --- | --- | --- | --- | --- | --- | --- | --- | --- |
| hotlake_ucc_124538 | CY41DRAFT_0125 | Nucleic acid metabolism | DNA replication_ recombination_ and repair | DNA repair protein radc |  | 9.761608 | 0.2443369 | 0.3846645 | 0.6351949 | 0.5253013 | 0.6083656 |
| hotlake_ucc_124539 | CY41DRAFT_0132 | Nucleic acid metabolism | DNA replication_ recombination_ and repair | Nucleotidyltransferase/DNA polymerase involved in DNA repair |  | 800.375384 | -1.0727827 | 0.3176367 | -3.3773892 | 0.0007318 | 0.0019697 |
| hotlake_ucc_124540 | CY41DRAFT_0143 | Mobile and extrachromosomal element functions | Selfish genetic elements | IS3 family transposase |  | 138.723430 | 0.7620211 | 0.2151973 | 3.5410352 | 0.0003986 | 0.0011479 |
| hotlake_ucc_124541 | CY41DRAFT_0145 |  |  | transcriptional regulator-like protein |  | 99.795097 | -0.4921529 | 0.2333701 | -2.1088944 | 0.0349537 | 0.0614616 |
| hotlake_ucc_124542 | CY41DRAFT_0180 |  |  | hypothetical protein |  | 60.185987 | 0.0664750 | 0.3848789 | 0.1727166 | 0.8628742 | 0.8995962 |
| hotlake_ucc_124543 | CY41DRAFT_0217 |  |  | transposase |  | 36.230425 | 0.9260233 | 0.3489880 | 2.6534531 | 0.0079673 | 0.0170592 |

RNA seq data processing for Marinobacter HL-58; generate normalized counts in RPKM

dfcountData <- data.frame(raw.58, row.names = 1)
dfcolData <- data.frame(info.58, row.names = 1)

dds.58 <- DESeqDataSetFromMatrix(countData = dfcountData, colData = dfcolData, design = ~condition)

## Warning in DESeqDataSet(se, design = design, ignoreRank): some variables in
## design formula are characters, converting to factors

dds.58 <- DESeq(dds.58)

## estimating size factors

## estimating dispersions

## gene-wise dispersion estimates

## mean-dispersion relationship

## -- note: fitType='parametric', but the dispersion trend was not well captured by the
## function: y = a/x + b, and a local regression fit was automatically substituted.
## specify fitType='local' or 'mean' to avoid this message next time.

## final dispersion estimates

## fitting model and testing

notAllZero <- (rowSums(counts(dds.58)) > 0)
vsd <- varianceStabilizingTransformation(dds.58)

## -- note: fitType='parametric', but the dispersion trend was not well captured by the
## function: y = a/x + b, and a local regression fit was automatically substituted.
## specify fitType='local' or 'mean' to avoid this message next time.

HL58.norm.count <- data.frame(assay(vsd[notAllZero,]))
HL58.norm.count <- add_rownames(HL58.norm.count, "GeneID")

## Warning: Deprecated, use tibble::rownames_to_column() instead.

HL58.norm.count <- merge(an.58, HL58.norm.count, by = "GeneID")

write.table(HL58.norm.count, file="RNA_seq_outputs/HL58_Norm_Exp_Values_annotated.csv", quote=FALSE, sep=",", row.names=FALSE, col.names=TRUE)
kable(head(HL58.norm.count))

| GeneID | Acc_num | Main_Role | Subrole | Product | Gene | T6_HL58G | T7_HL58G | T8_HL58G | T9_HL58G | T10_HL58G | T11_HL58G | T12_HL58G | T13_HL58G | T14_HL58G | T15_HL58G | T21_HL58X | T22_HL58X | T23_HL58X | T24_HL58X |
| --- | --- | --- | --- | --- | --- | --- | --- | --- | --- | --- | --- | --- | --- | --- | --- | --- | --- | --- | --- |
| hotlake_ucc_124834 | CD01DRAFT_3221 | Glycan biosynthesis and metabolism | Peptidoglycan metabolism | UDP-N-acetylmuramate dehydrogenase |  | 11.3358256 | 11.1800462 | 11.5913774 | 12.6287175 | 12.2522154 | 10.6245245 | 11.458865 | 10.8227724 | 11.3105664 | 11.868815 | 10.837703 | 10.8722827 | 10.9260847 | 9.8385927 |
| hotlake_ucc_124835 | CD01DRAFT_3256 | Xenobiotics biodegradation and metabolism | Benzoate degradation | acetyl-CoA acetyltransferase |  | 0.2893289 | 0.0618485 | 0.2695364 | -0.2584539 | 0.0814818 | 1.6733887 | 3.072722 | 1.2447668 | 1.4060076 | 3.627405 | 2.325894 | 2.8275946 | 2.6694170 | 0.6611770 |
| hotlake_ucc_80896 | CD01DRAFT_0003 | Regulatory functions::Signal transduction::Unknown function | Enzymes of unknown specificity::Two-component systems::Taxis::Small molecule interactions | two component signal transduction system histidine kinase |  | -3.9207873 | -3.8945012 | -3.5862830 | -4.0033188 | -4.8527091 | -4.2900475 | -5.839016 | -5.7183508 | -5.5616983 | -6.397703 | -6.397703 | -6.3977031 | -6.3977031 | -4.6529033 |
| hotlake_ucc_80898 | CD01DRAFT_0005 |  |  | outer membrane porin |  | -0.3121074 | -1.1220738 | -0.0941957 | 1.3831640 | -0.9062989 | 0.7489158 | -1.807176 | -0.8968551 | 0.1051990 | -1.515550 | 7.121093 | 7.8805851 | 7.4294346 | 9.2445007 |
| hotlake_ucc_80900 | CD01DRAFT_0006 | Unknown function | Enzymes of unknown specificity | Diacylglycerol O-acyltransferase |  | 0.1854444 | 0.3224793 | 0.0633558 | 0.0108127 | -0.4184039 | 0.5333536 | 1.387705 | -0.2424177 | 0.3700958 | 1.415378 | 2.325894 | -0.3955064 | -0.7815176 | 0.3493803 |
| hotlake_ucc_80901 | CD01DRAFT_0007 |  |  | hypothetical protein |  | 7.5393210 | 7.6051410 | 7.1777729 | 8.2342630 | 7.5543323 | 10.3865789 | 8.438497 | 10.8655177 | 10.2467437 | 8.178904 | 10.794648 | 11.1480828 | 11.0841307 | 10.6393807 |

# HL58.norm.count %>% head
# # So this simple df includes gene annotations and is normalized...
# dds.58 %>% assay() %>% head
# # ...while this is the full object, without normalization. Just like last time
colData(dds.58)$condition %>% table

## .
## HL_48_58_G HL_48_58_X HL_58_G
## 5 4 5

more RNA seq data processing for Marinobacter HL-58; generate differential expression output for HL-58 glucose competition treatment HL-58 glucose axenic vs. HL-58/HL-48 glucose co-culture note that HL-58 does not grow on xylose; hence, differential expression cannot be analyzed for HL-58 xylose commensalism

HL58.diff.comp <- deseq_diff(dds.58, c("condition","HL_48_58_G","HL_58_G"), an.58)

write.table(HL58.diff.comp, file="RNA_seq_outputs/HL58.diff.comp.csv", quote=FALSE, sep=",", row.names=TRUE, col.names=TRUE)
kable(head(HL58.diff.comp))

| GeneID | Acc_num | Main_Role | Subrole | Product | Gene | baseMean | log2FoldChange | lfcSE | stat | pvalue | padj |
| --- | --- | --- | --- | --- | --- | --- | --- | --- | --- | --- | --- |
| hotlake_ucc_124834 | CD01DRAFT_3221 | Glycan biosynthesis and metabolism | Peptidoglycan metabolism | UDP-N-acetylmuramate dehydrogenase |  | 2542.987859 | -0.3681853 | 0.2064011 | -1.7838339 | 0.0744507 | 0.1183062 |
| hotlake_ucc_124835 | CD01DRAFT_3256 | Xenobiotics biodegradation and metabolism | Benzoate degradation | acetyl-CoA acetyltransferase |  | 100.671202 | 1.1086581 | 0.2105873 | 5.2646020 | 0.0000001 | 0.0000008 |
| hotlake_ucc_80896 | CD01DRAFT_0003 | Regulatory functions::Signal transduction::Unknown function | Enzymes of unknown specificity::Two-component systems::Taxis::Small molecule interactions | two component signal transduction system histidine kinase |  | 3.303432 | -2.8295761 | 0.6983407 | -4.0518561 | 0.0000508 | 0.0001784 |
| hotlake_ucc_80897 | CD01DRAFT_0004 |  |  | COG3287 family protein of unknown function |  | 0.000000 | NA | NA | NA | NA | NA |
| hotlake_ucc_80898 | CD01DRAFT_0005 |  |  | outer membrane porin |  | 247.862849 | -0.2903843 | 0.3103231 | -0.9357484 | 0.3494028 | 0.4339249 |
| hotlake_ucc_80900 | CD01DRAFT_0006 | Unknown function | Enzymes of unknown specificity | Diacylglycerol O-acyltransferase |  | 67.393844 | 0.3850037 | 0.1726139 | 2.2304326 | 0.0257187 | 0.0469052 |

generate differential expression output for HL-58 competition over commensalism HL-58/HL-48 xylose co-culture (cmns) vs. HL-58/HL-48 glucose co-culture (comp)

# new graph comparing two treatments
HL58.diff.coculture <- deseq_diff(dds.58, c("condition","HL_48_58_G","HL_48_58_X"), an.58)

write.table(HL58.diff.coculture, file="RNA_seq_outputs/HL58.diff.coculture.csv", quote=FALSE, sep=",", row.names=TRUE, col.names=TRUE)
kable(head(HL58.diff.coculture))

| GeneID | Acc_num | Main_Role | Subrole | Product | Gene | baseMean | log2FoldChange | lfcSE | stat | pvalue | padj |
| --- | --- | --- | --- | --- | --- | --- | --- | --- | --- | --- | --- |
| hotlake_ucc_124834 | CD01DRAFT_3221 | Glycan biosynthesis and metabolism | Peptidoglycan metabolism | UDP-N-acetylmuramate dehydrogenase |  | 2542.987859 | 0.3579844 | 0.2244110 | 1.5952182 | 0.1106635 | 0.1751276 |
| hotlake_ucc_124835 | CD01DRAFT_3256 | Xenobiotics biodegradation and metabolism | Benzoate degradation | acetyl-CoA acetyltransferase |  | 100.671202 | 0.1746891 | 0.2913066 | 0.5996743 | 0.5487233 | 0.6405866 |
| hotlake_ucc_80896 | CD01DRAFT_0003 | Regulatory functions::Signal transduction::Unknown function | Enzymes of unknown specificity::Two-component systems::Taxis::Small molecule interactions | two component signal transduction system histidine kinase |  | 3.303432 | -1.3303797 | 1.2096676 | -1.0997895 | 0.2714238 | 0.3704850 |
| hotlake_ucc_80897 | CD01DRAFT_0004 |  |  | COG3287 family protein of unknown function |  | 0.000000 | NA | NA | NA | NA | NA |
| hotlake_ucc_80898 | CD01DRAFT_0005 |  |  | outer membrane porin |  | 247.862849 | -4.0592778 | 0.3342160 | -12.1456716 | 0.0000000 | 0.0000000 |
| hotlake_ucc_80900 | CD01DRAFT_0006 | Unknown function | Enzymes of unknown specificity | Diacylglycerol O-acyltransferase |  | 67.393844 | 0.0106930 | 0.2899656 | 0.0368768 | 0.9705832 | 0.9780839 |

generate differential expression output for HL-58 xylose commensalism over glucose axenic HL-58 glucose axenic vs. HL-58/HL-48 xylose co-culture

Because HL-58 does not grow on xylose, but can survive in the presence of HL-48, this is used as a proxy for the impossible HL_58_X. Note that two treatments are applied (different substrate and introduction of HL-48).

# new graph control to two treatments
HL58.diff.proxy <- deseq_diff(dds.58, c("condition","HL_48_58_X","HL_58_G"), an.58)

write.table(HL58.diff.proxy, file="RNA_seq_outputs/HL58.diff.proxy.csv", quote=FALSE, sep=",", row.names=TRUE, col.names=TRUE)
kable(head(HL58.diff.proxy))

| GeneID | Acc_num | Main_Role | Subrole | Product | Gene | baseMean | log2FoldChange | lfcSE | stat | pvalue | padj |
| --- | --- | --- | --- | --- | --- | --- | --- | --- | --- | --- | --- |
| hotlake_ucc_124834 | CD01DRAFT_3221 | Glycan biosynthesis and metabolism | Peptidoglycan metabolism | UDP-N-acetylmuramate dehydrogenase |  | 2542.987859 | -0.7261697 | 0.2241244 | -3.240030 | 0.0011952 | 0.0034253 |
| hotlake_ucc_124835 | CD01DRAFT_3256 | Xenobiotics biodegradation and metabolism | Benzoate degradation | acetyl-CoA acetyltransferase |  | 100.671202 | 0.9339691 | 0.2889583 | 3.232193 | 0.0012284 | 0.0035027 |
| hotlake_ucc_80896 | CD01DRAFT_0003 | Regulatory functions::Signal transduction::Unknown function | Enzymes of unknown specificity::Two-component systems::Taxis::Small molecule interactions | two component signal transduction system histidine kinase |  | 3.303432 | -1.4991964 | 1.0699322 | -1.401207 | 0.1611522 | 0.2438791 |
| hotlake_ucc_80897 | CD01DRAFT_0004 |  |  | COG3287 family protein of unknown function |  | 0.000000 | NA | NA | NA | NA | NA |
| hotlake_ucc_80898 | CD01DRAFT_0005 |  |  | outer membrane porin |  | 247.862849 | 3.7688935 | 0.3242340 | 11.623992 | 0.0000000 | 0.0000000 |
| hotlake_ucc_80900 | CD01DRAFT_0006 | Unknown function | Enzymes of unknown specificity | Diacylglycerol O-acyltransferase |  | 67.393844 | 0.3743107 | 0.2844672 | 1.315831 | 0.1882308 | 0.2780080 |

## Plot differentially expressed genes

setup volcano plot for HL48 glucose competition this analysis barrows ideas/code from a previously described example source <https://twbattaglia.github.io/2016/12/17/volcano-plot/>

add_color_cutoff <- function(df, pvalue_cutoff = -log10(0.05), up_name, down_name, default_name = "None"){
 # This function is hard-coded to our data. It's not meant to be a general function.
 df$color <- default_name
 df$color[df$lfc > 0 & df$pvalue > pvalue_cutoff] <- up_name
 df$color[df$lfc < 0 & df$pvalue > pvalue_cutoff] <- down_name

 return(df)
}

# https://stackoverflow.com/questions/7367138/text-wrap-for-plot-titles
wrap_strings <- function(x, goal_width = 40){
 as.character(sapply(x,FUN=function(x){
 if(is.na(x)){return("")}
 if(nchar(x) == 0){return("")}
 print((nchar(x) / (ceiling(nchar(x)/goal_width))))
 paste(strwrap(x, width = (nchar(x) / (ceiling(nchar(x)/goal_width)))), collapse=" \n")
 }))
}

# Test:
c("test", "", NA) %>% wrap_strings()

## [1] 4

## [1] "test" "" ""

# Also use it on full gp.labels
gp.labels$Product <- wrap_strings(gp.labels$Product)

## [1] 29
## [1] 33
## [1] 31.5
## [1] 31.5
## [1] 18
## [1] 33
## [1] 21
## [1] 24.5
## [1] 24.5
## [1] 26
## [1] 31.5
## [1] 40
## [1] 33
## [1] 25
## [1] 25.5
## [1] 26.5
## [1] 40
## [1] 21
## [1] 23
## [1] 22.5
## [1] 20.5
## [1] 38
## [1] 23
## [1] 37
## [1] 35
## [1] 35

plot_v <- function(df, df.labels){
 # This function is hard-coded to our data. It's not meant to be a general function.
 plot <- ggplot(df, aes(x = lfc, y = pvalue))
 return(plot +
 #geom_vline(xintercept = 0, color = "black") + # add line at 0
 geom_vline(xintercept = c(-log2(2),log2(2)), color = "grey40") + # Add cutoffs
 geom_hline(yintercept = -log10(0.05), color = "grey40") + # we put our pvalue cutoff in here
 geom_point(aes(color = factor(color)), size = 2, alpha = 0.5, na.rm = TRUE) +
 theme(legend.position = "none") + # remove legend
 # We let's add these manually so they match the different graph
 #annotate("text", x = -2, y = 0, label = "Axenic", size = 5, color = "black") + # add Untreated text
 #annotate("text", x = 2, y = 0, label = "Co-culture", size = 5, color = "red") + # add Treated text
 #xlab(expression(log[2]("Co-culture" / "Axenic"))) + # x-axis label
 ylab(expression(-log[10]("adjusted p-value"))) + # y-axis label
 scale_y_continuous(trans = "log1p") + #transform the y-axis
 scale_color_manual(values = c("Co-culture" = viridis(10, option = "C")[8],
 "Axenic" = viridis(10, option = "C")[1],
 "None" = "grey")) + # We could add new colors here for Commensalism and Competition plots.
 geom_text_repel(data = df.labels, lineheight = 0.8,
 mapping = aes(label = Product), min.segment.length = unit(0, "lines")
 ,box.padding = unit(0.2, "lines"), point.padding = unit(0.2, "lines")
 )
 )
}

### HL-48 response to glucose competition

Fig2-A, Colored by directionality


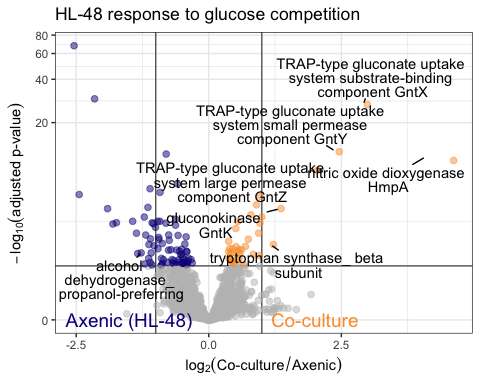


### HL-48 response to xylose commensalism

Colored by directionality


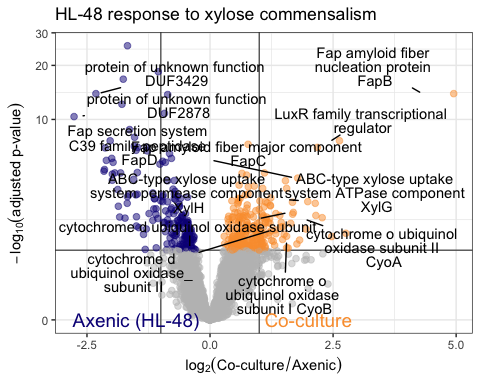


### HL-58 response to glucose competition

Colored by directionality


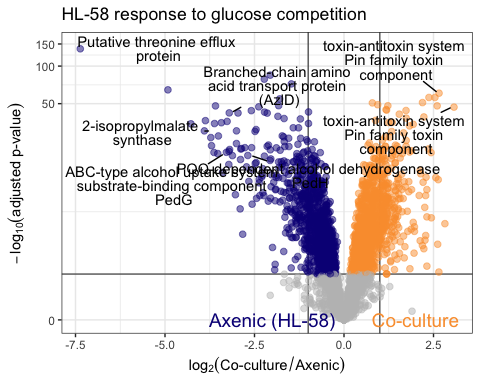


### Three new volcano plots

## [1] 23
## [1] 32.5
## [1] 31
## [1] 19
## [1] 38.5
## [1] 20
## [1] 34
## [1] 35
## [1] 22
## [1] 34.5

## Warning: Removed 2 rows containing missing values (geom_text_repel).


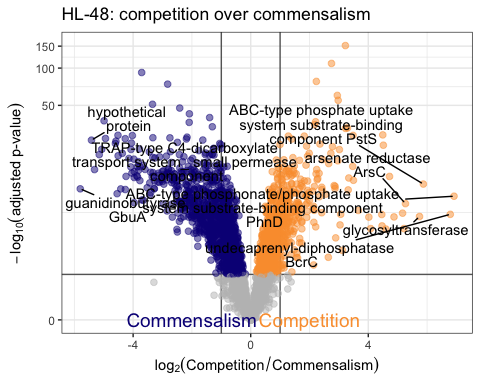


## [1] 24
## [1] 34
## [1] 19
## [1] 23
## [1] 27
## [1] 16
## [1] 26.5
## [1] 32.5
## [1] 23
## [1] 37


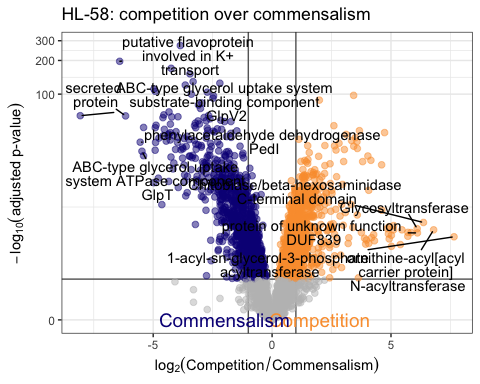


## [1] 38
## [1] 38.5
## [1] 26.5
## [1] 32.5
## [1] 23
## [1] 24
## [1] 22
## [1] 19
## [1] 23
## [1] 27


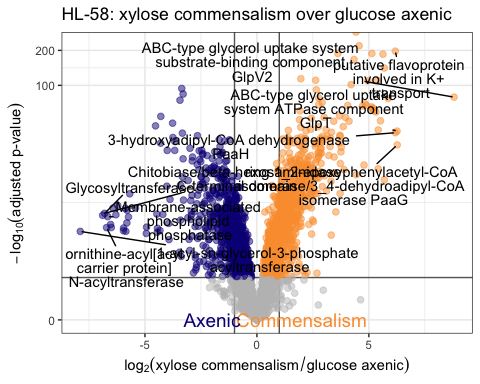


# Functional Enrichment (FE)

Dot plots!

This section applies filters to differentially expressed genes and calculates those gene function categories that are statistically enriched from the genome of each species

#trim differentially expressed genes by a pvalue cutoff p-adjusted <= 0.05
HL48.diff.comp.filt <- HL48.diff.comp %>% subset(padj <= 0.05)
HL48.diff.cmns.filt <- HL48.diff.cmns %>% subset(padj <= 0.05)
HL58.diff.comp.filt <- HL58.diff.comp %>% subset(padj <= 0.05)

# fold changes that are significant
HL58.diff.comp.filt$log2FoldChange %>% qplot() + geom_histogram()

## `stat_bin()` using `bins = 30`. Pick better value with `binwidth`.
## `stat_bin()` using `bins = 30`. Pick better value with `binwidth`.


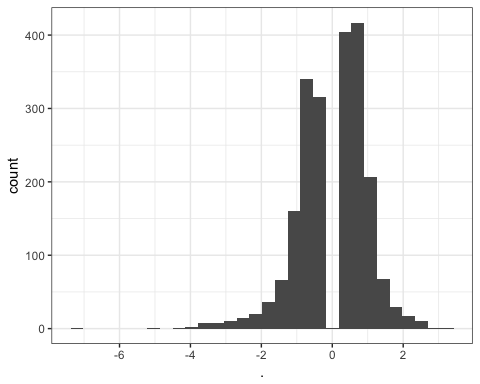


#trim differentially expressed genes by fold change; FC greater than 2 (< log2 = -1 or > log2 = 1)
HL48.diff.comp.filtU <- HL48.diff.comp.filt %>% subset(log2FoldChange >= 1)
HL48.diff.cmns.filtU <- HL48.diff.cmns.filt %>% subset(log2FoldChange >= 1)
HL58.diff.comp.filtU <- HL58.diff.comp.filt %>% subset(log2FoldChange >= 1)
# HL48.diff.comp.filt <- HL48.diff.comp.filt %>% subset(abs(log2FoldChange) >= 1)
# HL48.diff.cmns.filt <- HL48.diff.cmns.filt %>% subset(abs(log2FoldChange) >= 1)
# HL58.diff.comp.filt <- HL58.diff.comp.filt %>% subset(abs(log2FoldChange) >= 1)
HL48.diff.comp.filtD <- HL48.diff.comp.filt %>% subset(log2FoldChange <= -1)
HL48.diff.cmns.filtD <- HL48.diff.cmns.filt %>% subset(log2FoldChange <= -1)
HL58.diff.comp.filtD <- HL58.diff.comp.filt %>% subset(log2FoldChange <= -1)

# HL58.diff.comp.filtU$log2FoldChange %>% qplot() + geom_histogram() # all up
# HL58.diff.comp.filtD$log2FoldChange %>% qplot() + geom_histogram() # all down

# fold changes that are significant and OVER 2 (but not under). So this capture enrichment only.
HL58.diff.comp.filt$log2FoldChange %>% qplot() + geom_histogram()

## `stat_bin()` using `bins = 30`. Pick better value with `binwidth`.
## `stat_bin()` using `bins = 30`. Pick better value with `binwidth`.


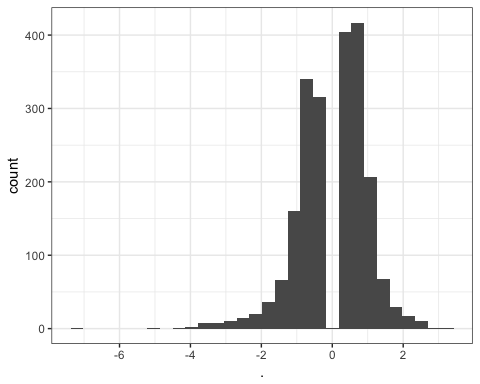


#prepare input files for FE
HL48.comp.FE.U <- HL48.diff.comp.filtU %>% select(GeneID) %>% data.frame(., ModuleID = "HL_48_58_G_v_HL_48_G")
HL48.cmns.FE.U <- HL48.diff.cmns.filtU %>% select(GeneID) %>% data.frame(., ModuleID = "HL_48_58_X_v_HL_48_X")
HL58.comp.FE.U <- HL58.diff.comp.filtU %>% select(GeneID) %>% data.frame(., ModuleID = "HL_48_58_G_v_HL_58_G")

HL48.comp.FE.D <- HL48.diff.comp.filtD %>% select(GeneID) %>% data.frame(., ModuleID = "HL_48_58_G_v_HL_48_G")
HL48.cmns.FE.D <- HL48.diff.cmns.filtD %>% select(GeneID) %>% data.frame(., ModuleID = "HL_48_58_X_v_HL_48_X")
HL58.comp.FE.D <- HL58.diff.comp.filtD %>% select(GeneID) %>% data.frame(., ModuleID = "HL_48_58_G_v_HL_58_G")

FE calculations of main role categories

#first, set up the subrole FE function
#input x is a FE input e.g. HL48.comp.FE
#input y is an annotation file e.g. an.48
mainroleeModuleEnrichment <- function(x,y)
{
 y[grepl('::', y$Main_Role), 'Main_Role'] <- 'Ambiguous_Function'
 fModuleData <- x
 fAnnotData <- y[, c("GeneID", "Main_Role")]
 colnames(fAnnotData) <- c("GeneID", "Main_Role")
 uniqueFunCats <- unique(fAnnotData[c("Main_Role")])
 Main_Role <- unique(fAnnotData$Main_Role)
 modules <- unique(fModuleData$ModuleID)
 numGenesInGenome <- nrow(fAnnotData)
 outputData <- NULL
 for (mID in modules)
 {
 genesInSet <- fModuleData[fModuleData$ModuleID == mID, "GeneID"]
 numGenesInSet <- length(genesInSet)
 for (i in 1:nrow(uniqueFunCats))
 {
 Main_Role <- uniqueFunCats[i, "Main_Role"]
 genesInGenomeWithAnnot <- fAnnotData[fAnnotData$Main_Role == Main_Role, "GeneID"]
 numGenesInGenomeWithAnnot <- length(genesInGenomeWithAnnot)
 numGenesInSetWithAnnot <- length(intersect(genesInSet, genesInGenomeWithAnnot))
 #=====================================================================================
 # Run Fisher's exact test
 counts <- matrix(c(numGenesInSetWithAnnot, numGenesInSet-numGenesInSetWithAnnot,
 numGenesInGenomeWithAnnot, numGenesInGenome-numGenesInGenomeWithAnnot), nrow=2)
 res <- fisher.test(counts)
 if (res$p.value <= 0.05)
 {
 pModule <- numGenesInSetWithAnnot/numGenesInSet
 pGenome <- numGenesInGenomeWithAnnot/numGenesInGenome
 ratio <- pModule/pGenome
 if (pModule > pGenome)
 {
 outputData <- rbind(outputData, cbind(ModuleID=mID, Main_Role=Main_Role, PVal=res$p.value, Ratio=ratio, PercentageInModule=pModule, PercentageInGenome=pGenome))
 }
 }
 }
 }
 outputData <- data.frame(outputData)
 outputData
}

#mainrolefunctial enrichment function using HL48.comp.FE and an.48 as x and y inputs

HL48.comp.MR.FE.U <- mainroleeModuleEnrichment(HL48.comp.FE.U, an.48)
HL48.comp.MR.FE.U$Treatment <- "HL-48 Glucose\nCompetition"
HL48.comp.MR.FE.U$dir <- "Increase"

#mainrolefunctial enrichment function using HL48.cmns.FE and an.48 as x and y inputs
HL48.cmns.MR.FE.U <- mainroleeModuleEnrichment(HL48.cmns.FE.U, an.48)
HL48.cmns.MR.FE.U$Treatment <- "HL-48 Xylose\nCommensalism"
HL48.cmns.MR.FE.U$dir <- "Increase"

#mainrolefunctial enrichment function using HL58.comp.FE and an.58 as x and y inputs
HL58.comp.MR.FE.U <- mainroleeModuleEnrichment(HL58.comp.FE.U, an.58)
HL58.comp.MR.FE.U$Treatment <- 'HL-58 Glucose\nCompetition'
HL58.comp.MR.FE.U$dir <- "Increase"


# matching set for reduced (D Down) enrichment
HL48.comp.MR.FE.D <- mainroleeModuleEnrichment(HL48.comp.FE.D, an.48)
HL48.comp.MR.FE.D$Treatment <- 'HL-48 Glucose\nCompetition'
HL48.comp.MR.FE.D$dir <- "Decrease"
# Empty?

HL48.cmns.MR.FE.D <- mainroleeModuleEnrichment(HL48.cmns.FE.D, an.48)
HL48.cmns.MR.FE.D$Treatment <- 'HL-48 Xylose\nCommensalism'
HL48.cmns.MR.FE.D$dir <- "Decrease"

HL58.comp.MR.FE.D <- mainroleeModuleEnrichment(HL58.comp.FE.D, an.58)
HL58.comp.MR.FE.D$Treatment <- 'HL-58 Glucose\nCompetition'
HL58.comp.MR.FE.D$dir <- "Decrease"


#pull it together
MR.FE <- rbind(HL48.comp.MR.FE.U, HL48.cmns.MR.FE.U, HL58.comp.MR.FE.U,
 HL48.comp.MR.FE.D, # This is the empty data frame
 HL48.cmns.MR.FE.D, HL58.comp.MR.FE.D)
kable(MR.FE, caption = "Functional enrichment of main role gene categories")

Functional enrichment of main role gene categories

| ModuleID | Main_Role | PVal | Ratio | PercentageInModule | PercentageInGenome | Treatment | dir |
| --- | --- | --- | --- | --- | --- | --- | --- |
| HL_48_58_G_v_HL_48_G | Transport and binding proteins | 0.0247849323290425 | 4.3644578313253 | 0.428571428571429 | 0.0981958000591541 | HL-48 Glucose |  |
| Competition Increase |  |  |  |  |  |  |  |
| HL_48_58_X_v_HL_48_X | Cell structure_ growth_ and death | 0.000621398637458999 | 5.10504745470233 | 0.0921052631578947 | 0.018041999408459 | HL-48 Xylose |  |
| Commensalism Increase |  |  |  |  |  |  |  |
| HL_48_58_X_v_HL_48_X | Energy metabolism | 0.00765840961416274 | 3.60704125177809 | 0.0789473684210526 | 0.0218870156758355 | HL-48 Xylose |  |
| Commensalism Increase |  |  |  |  |  |  |  |
| HL_48_58_X_v_HL_48_X | Translation | 0.000125291419464542 | 3.68166969147005 | 0.157894736842105 | 0.0428867199053534 | HL-48 Xylose |  |
| Commensalism Increase |  |  |  |  |  |  |  |
| HL_48_58_X_v_HL_48_X | Transcription | 0.00116854340692983 | 9.8859649122807 | 0.0526315789473684 | 0.00532386867790594 | HL-48 Xylose |  |
| Commensalism Increase |  |  |  |  |  |  |  |
| HL_48_58_G_v_HL_58_G | Intracellular trafficking_ assembly_ and processing | 0.0264067225592166 | 1.82752593907784 | 0.0631970260223048 | 0.0345806451612903 | HL-58 Glucose |  |
| Competition Increase |  |  |  |  |  |  |  |
| HL_48_58_G_v_HL_58_G | Transport and binding proteins | 0.0137794066704247 | 1.618562298985 | 0.111524163568773 | 0.0689032258064516 | HL-58 Glucose |  |
| Competition Increase |  |  |  |  |  |  |  |
| HL_48_58_G_v_HL_58_G | Carbohydrate metabolism | 0.0391890775576213 | 1.92069392812887 | 0.0446096654275093 | 0.0232258064516129 | HL-58 Glucose |  |
| Competition Increase |  |  |  |  |  |  |  |
| HL_48_58_G_v_HL_58_G | Nucleic acid metabolism | 0.00706118571573953 | 2.26598721857901 | 0.0520446096654275 | 0.0229677419354839 | HL-58 Glucose |  |
| Competition Increase |  |  |  |  |  |  |  |
| HL_48_58_G_v_HL_48_G |  | 0.0270972329550317 | 1.71859237536657 | 0.52 | 0.302573203194321 | HL-48 Glucose |  |
| Competition Decrease |  |  |  |  |  |  |  |
| HL_48_58_X_v_HL_48_X | Metabolism of other amino acids and amines | 0.0244267716725998 | 9.11320754716981 | 0.0377358490566038 | 0.0041407867494824 | HL-48 Xylose |  |
| Commensalism Decrease |  |  |  |  |  |  |  |
| HL_48_58_G_v_HL_58_G |  | 6.67985051333306e-08 | 1.48494842015754 | 0.515037593984962 | 0.346838709677419 | HL-58 Glucose |  |
| Competition Decrease |  |  |  |  |  |  |  |
| HL_48_58_G_v_HL_58_G | Cell motility and adherance | 0.0303716770237368 | 2.33082706766917 | 0.0300751879699248 | 0.0129032258064516 | HL-58 Glucose |  |
| Competition Decrease |  |  |  |  |  |  |  |

## Plot main role functional enrichment results

MR <- data.frame(dir = MR.FE$dir, Treatment = MR.FE$Treatment, Main_Role = MR.FE$Main_Role,
 Ratio = as.numeric(MR.FE$Ratio), PercentageInModule = 100*(as.numeric(MR.FE$PercentageInModule)))

MR$Ratio %>% summary

## Min. 1st Qu. Median Mean 3rd Qu. Max.
## 1.485 1.828 2.331 3.763 4.364 9.886

g.MR.FE <- ggplot(MR, aes(x = Treatment, y = Main_Role, size = Ratio, fill = PercentageInModule))
g.MR.FE <- g.MR.FE +
 #geom_point(shape = 21, colour = "#000000", fill = "#40b8d0") +
 geom_point(shape = 21) +
 facet_grid(~dir) +
 ggtitle("Functional enrichment of gene main roles") +
 labs(x = "Treatment", y = "")
g.MR.FE


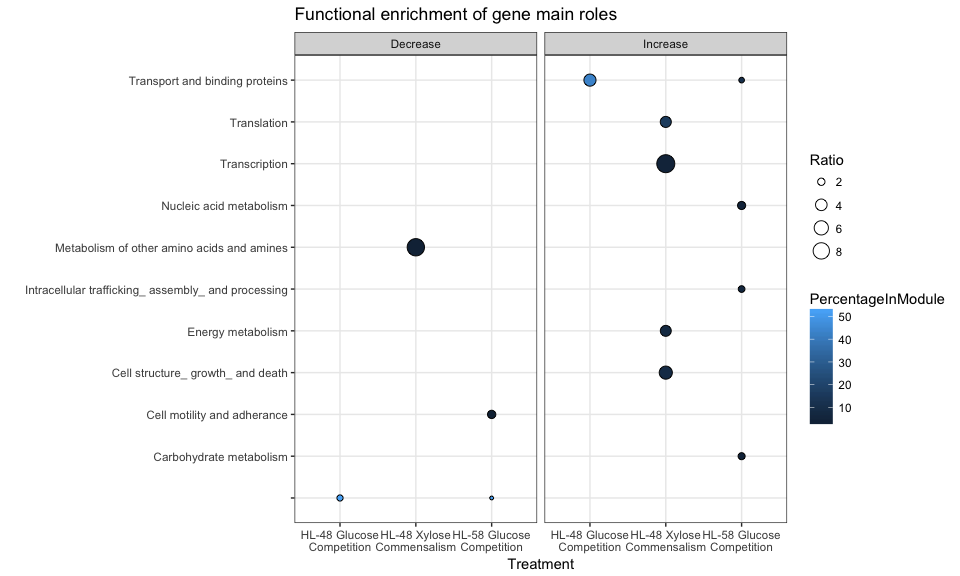


FE calculations on main role categories

#first, set up the subrole FE function
#input x is a FE input e.g. HL48.comp.FE
#input y is an annotation file e.g. an.48
subroleModuleEnrichment <- function(x,y)
{
 y[grepl('::', y$Subrole), 'Subrole'] <- 'Ambiguous_Function'
 fModuleData <- x
 fAnnotData <- y[, c("GeneID", "Subrole")]
 colnames(fAnnotData) <- c("GeneID", "Subrole")
 uniqueFunCats <- unique(fAnnotData[c("Subrole")])
 subRole <- unique(fAnnotData$Subrole)
 modules <- unique(fModuleData$ModuleID)
 numGenesInGenome <- nrow(fAnnotData)
 outputData <- NULL
 for (mID in modules)
 {
 genesInSet <- fModuleData[fModuleData$ModuleID == mID, "GeneID"]
 numGenesInSet <- length(genesInSet)
 for (i in 1:nrow(uniqueFunCats))
 {
 subRole <- uniqueFunCats[i, "Subrole"]
 genesInGenomeWithAnnot <- fAnnotData[fAnnotData$Subrole == subRole, "GeneID"]
 numGenesInGenomeWithAnnot <- length(genesInGenomeWithAnnot)
 numGenesInSetWithAnnot <- length(intersect(genesInSet, genesInGenomeWithAnnot))
 #=====================================================================================
 # Run Fisher's exact test
 counts <- matrix(c(numGenesInSetWithAnnot, numGenesInSet-numGenesInSetWithAnnot,
 numGenesInGenomeWithAnnot, numGenesInGenome-numGenesInGenomeWithAnnot), nrow=2)
 res <- fisher.test(counts)
 if (res$p.value <= 0.05)
 {
 pModule <- numGenesInSetWithAnnot/numGenesInSet
 pGenome <- numGenesInGenomeWithAnnot/numGenesInGenome
 ratio <- pModule/pGenome
 if (pModule > pGenome)
 {
 outputData <- rbind(outputData, cbind(ModuleID=mID, Subrole=subRole, PVal=res$p.value, Ratio=ratio, PercentageInModule=pModule, PercentageInGenome=pGenome))
 }
 }
 }
 }
 outputData <- data.frame(outputData)
 outputData
}

#subrolefunctial enrichment function using HL48.comp.FE and an.48 as x and y inputs
HL48.comp.SR.FE.U <- subroleModuleEnrichment(HL48.comp.FE.U, an.48)
HL48.comp.SR.FE.U$Treatment <- 'HL-48 Glucose\nCompetition'
HL48.comp.SR.FE.U$dir <- "Increase"

#subrolefunctial enrichment function using HL48.cmns.FE and an.48 as x and y inputs
HL48.cmns.SR.FE.U <- subroleModuleEnrichment(HL48.cmns.FE.U, an.48)
HL48.cmns.SR.FE.U$Treatment <- 'HL-48 Xylose\nCommensalism'
HL48.cmns.SR.FE.U$dir <- "Increase"

#subrolefunctial enrichment function using HL58.comp.FE and an.58 as x and y inputs
HL58.comp.SR.FE.U <- subroleModuleEnrichment(HL58.comp.FE.U, an.58)
HL58.comp.SR.FE.U$Treatment <- 'HL-58 Glucose\nCompetition'
HL58.comp.SR.FE.U$dir <- "Increase"


# matching set for reduced (D Down) enrichment
HL48.comp.SR.FE.D <- subroleModuleEnrichment(HL48.comp.FE.D, an.48)
HL48.comp.SR.FE.D$Treatment <- 'HL-48 Glucose\nCompetition'
HL48.comp.SR.FE.D$dir <- "Decrease"

HL48.cmns.SR.FE.D <- subroleModuleEnrichment(HL48.cmns.FE.D, an.48)
HL48.cmns.SR.FE.D$Treatment <- 'HL-48 Xylose\nCommensalism'
HL48.cmns.SR.FE.D$dir <- "Decrease"

HL58.comp.SR.FE.D <- subroleModuleEnrichment(HL58.comp.FE.D, an.58)
HL58.comp.SR.FE.D$Treatment <- 'HL-58 Glucose\nCompetition'
HL58.comp.SR.FE.D$dir <- "Decrease"


#pull it together
SR.FE <- rbind(HL48.comp.SR.FE.U, HL48.cmns.SR.FE.U, HL58.comp.SR.FE.U,
 HL48.comp.SR.FE.D, HL48.cmns.SR.FE.D, HL58.comp.SR.FE.D)
kable(SR.FE, caption = "Functional enrichment of subrole gene categories")

Functional enrichment of subrole gene categories

| ModuleID | Subrole | PVal | Ratio | PercentageInModule | PercentageInGenome | Treatment | dir |
| --- | --- | --- | --- | --- | --- | --- | --- |
| HL_48_58_G_v_HL_48_G | Carbohydrates_ organic alcohols_ and acids | 0.00248325134199355 | 26.8333333333333 | 0.285714285714286 | 0.0106477373558119 | HL-48 Glucose |  |
| Competition Increase |  |  |  |  |  |  |  |
| HL_48_58_G_v_HL_48_G | Phenylalanine_ tyrosine and tryptophan biosynthesis | 0.0366344810198446 | 28.4117647058824 | 0.142857142857143 | 0.00502809819580006 | HL-48 Glucose |  |
| Competition Increase |  |  |  |  |  |  |  |
| HL_48_58_G_v_HL_48_G | Pyruvate metabolism | 0.0184637316007577 | 60.375 | 0.142857142857143 | 0.00236616385684709 | HL-48 Glucose |  |
| Competition Increase |  |  |  |  |  |  |  |
| HL_48_58_X_v_HL_48_X | Protein export_ secretion_ and sorting | 0.016590769967938 | 6.06638755980861 | 0.0394736842105263 | 0.00650695060632949 | HL-48 Xylose |  |
| Commensalism Increase |  |  |  |  |  |  |  |
| HL_48_58_X_v_HL_48_X | Cell division | 0.000522576883149083 | 12.7105263157895 | 0.0526315789473684 | 0.0041407867494824 | HL-48 Xylose |  |
| Commensalism Increase |  |  |  |  |  |  |  |
| HL_48_58_X_v_HL_48_X | Transcription factors | 0.0469103929027543 | 6.35526315789474 | 0.0263157894736842 | 0.0041407867494824 | HL-48 Xylose |  |
| Commensalism Increase |  |  |  |  |  |  |  |
| HL_48_58_X_v_HL_48_X | Oxidative phosphorylation | 0.00128086052095729 | 6.74043062200957 | 0.0657894736842105 | 0.00976042590949423 | HL-48 Xylose |  |
| Commensalism Increase |  |  |  |  |  |  |  |
| HL_48_58_X_v_HL_48_X | Chromosome and assoicated proteins | 0.0272987844560061 | 8.89736842105263 | 0.0263157894736842 | 0.00295770482105886 | HL-48 Xylose |  |
| Commensalism Increase |  |  |  |  |  |  |  |
| HL_48_58_X_v_HL_48_X | Ribosomal proteins: synthesis and modification | 8.62947452254726e-05 | 6.0321141837645 | 0.105263157894737 | 0.0174504584442473 | HL-48 Xylose |  |
| Commensalism Increase |  |  |  |  |  |  |  |
| HL_48_58_X_v_HL_48_X | RNA polymerase | 0.00675724604672468 | 22.2434210526316 | 0.0263157894736842 | 0.00118308192842354 | HL-48 Xylose |  |
| Commensalism Increase |  |  |  |  |  |  |  |
| HL_48_58_G_v_HL_58_G | Peptidoglycan metabolism | 0.00174061263396245 | 4.1157727031333 | 0.0297397769516729 | 0.0072258064516129 | HL-58 Glucose |  |
| Competition Increase |  |  |  |  |  |  |  |
| HL_48_58_G_v_HL_58_G | Protein export_ secretion_ and sorting | 0.0369465840600951 | 2.40086741016109 | 0.0260223048327138 | 0.0108387096774194 | HL-58 Glucose |  |
| Competition Increase |  |  |  |  |  |  |  |
| HL_48_58_G_v_HL_58_G | Pentose phosphate pathway | 0.0077778747775878 | 6.40231309376291 | 0.0148698884758364 | 0.00232258064516129 | HL-58 Glucose |  |
| Competition Increase |  |  |  |  |  |  |  |
| HL_48_58_G_v_HL_58_G | Amino acids_ peptides and amines | 0.0257608076700826 | 4.1157727031333 | 0.0148698884758364 | 0.00361290322580645 | HL-58 Glucose |  |
| Competition Increase |  |  |  |  |  |  |  |
| HL_48_58_G_v_HL_58_G | Citrate cycle (TCA cycle) | 0.0141938924524084 | 4.00144568360182 | 0.0185873605947955 | 0.00464516129032258 | HL-58 Glucose |  |
| Competition Increase |  |  |  |  |  |  |  |
| HL_48_58_G_v_HL_48_G |  | 0.0270972329550317 | 1.71859237536657 | 0.52 | 0.302573203194321 | HL-48 Glucose |  |
| Competition Decrease |  |  |  |  |  |  |  |
| HL_48_58_G_v_HL_48_G | General | 0.0372163936778414 | 3.14511627906977 | 0.16 | 0.0508725229222124 | HL-48 Glucose |  |
| Competition Decrease |  |  |  |  |  |  |  |
| HL_48_58_X_v_HL_48_X | Small molecule interactions | 0.0277507186204185 | 4.78443396226415 | 0.0566037735849057 | 0.0118308192842354 | HL-48 Xylose |  |
| Commensalism Decrease |  |  |  |  |  |  |  |
| HL_48_58_X_v_HL_48_X | Alanine_ aspartate and glutamate metabolism | 0.016352582304608 | 11.598627787307 | 0.0377358490566038 | 0.00325347530316474 | HL-48 Xylose |  |
| Commensalism Decrease |  |  |  |  |  |  |  |
| HL_48_58_X_v_HL_48_X | aliphatic and aromatic amines | 0.0456038265878301 | 31.8962264150943 | 0.0188679245283019 | 0.000591540964211772 | HL-48 Xylose |  |
| Commensalism Decrease |  |  |  |  |  |  |  |
| HL_48_58_G_v_HL_58_G |  | 6.67985051333306e-08 | 1.48494842015754 | 0.515037593984962 | 0.346838709677419 | HL-58 Glucose |  |
| Competition Decrease |  |  |  |  |  |  |  |
| HL_48_58_G_v_HL_58_G | Ubiquinone and other terpenoid-quinone biosynthesis | 0.0373922695734922 | 4.85588972431078 | 0.0112781954887218 | 0.00232258064516129 | HL-58 Glucose |  |
| Competition Decrease |  |  |  |  |  |  |  |
| HL_48_58_G_v_HL_58_G | Secretion systems | 0.0203596330863447 | 4.48235974551764 | 0.0150375939849624 | 0.00335483870967742 | HL-58 Glucose |  |
| Competition Decrease |  |  |  |  |  |  |  |
| HL_48_58_G_v_HL_58_G | Flagella complex and associated proteins | 0.0103865233371454 | 2.91353383458647 | 0.0300751879699248 | 0.0103225806451613 | HL-58 Glucose |  |
| Competition Decrease |  |  |  |  |  |  |  |
| HL_48_58_G_v_HL_58_G | Iron sulfur clusters | 0.000883817191067721 | 9.10479323308271 | 0.018796992481203 | 0.00206451612903226 | HL-58 Glucose |  |
| Competition Decrease |  |  |  |  |  |  |  |

## Plot subrole functional enrichment results

SR <- data.frame(dir = SR.FE$dir, Treatment = SR.FE$Treatment, Subrole = SR.FE$Subrole,
 Ratio = as.numeric(SR.FE$Ratio), PercentageInModule = 100*(as.numeric(SR.FE$PercentageInModule)))

SR$Ratio %>% summary

## Min. 1st Qu. Median Mean 3rd Qu. Max.
## 1.485 4.116 6.066 11.267 11.599 60.375

g.SR.FE <- ggplot(SR, aes(x = Treatment, y = Subrole, size = Ratio, fill = PercentageInModule))
g.SR.FE <- g.SR.FE +
 facet_grid(~dir) +
 #geom_point(shape = 21, colour = "#000000", fill = "#40b8d0") +
 geom_point(shape = 21) +
 ggtitle("Functional enrichment of gene subroles") +
 labs(x = "", y = "")
g.SR.FE


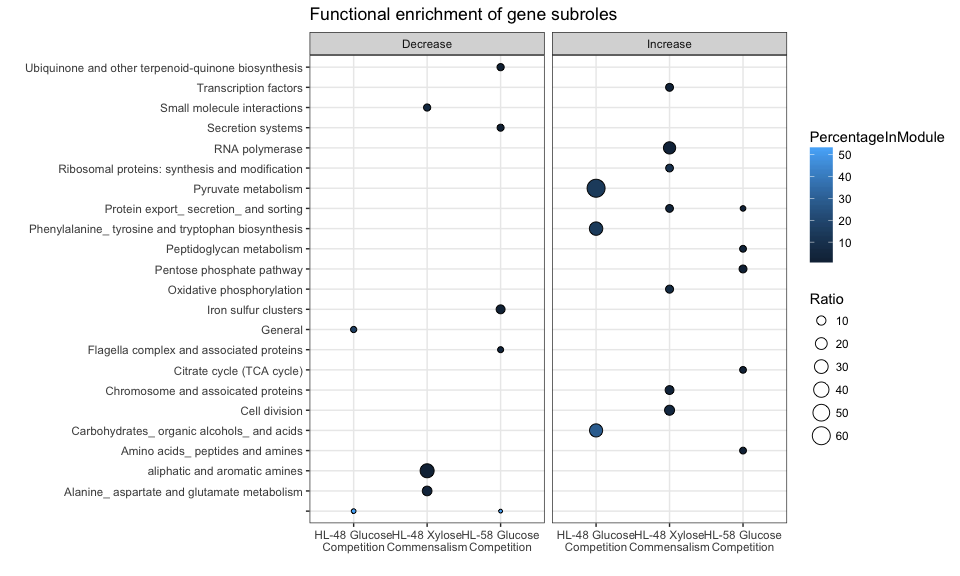


## Combined plot of main-role and subrole functional enrichment results

names(MR)[3] <- "Main Role"
names(SR)[3] <- "Subrole"

head(MR)

## dir Treatment
## 1 Increase HL-48 Glucose\nCompetition
## 2 Increase HL-48 Xylose\nCommensalism
## 3 Increase HL-48 Xylose\nCommensalism
## 4 Increase HL-48 Xylose\nCommensalism
## 5 Increase HL-48 Xylose\nCommensalism
## 6 Increase HL-58 Glucose\nCompetition
## Main Role Ratio
## 1 Transport and binding proteins 4.364458
## 2 Cell structure_ growth_ and death 5.105047
## 3 Energy metabolism 3.607041
## 4 Translation 3.681670
## 5 Transcription 9.885965
## 6 Intracellular trafficking_ assembly_ and processing 1.827526
## PercentageInModule
## 1 42.857143
## 2 9.210526
## 3 7.894737
## 4 15.789474
## 5 5.263158
## 6 6.319703

head(SR)

## dir Treatment
## 1 Increase HL-48 Glucose\nCompetition
## 2 Increase HL-48 Glucose\nCompetition
## 3 Increase HL-48 Glucose\nCompetition
## 4 Increase HL-48 Xylose\nCommensalism
## 5 Increase HL-48 Xylose\nCommensalism
## 6 Increase HL-48 Xylose\nCommensalism
## Subrole Ratio
## 1 Carbohydrates_ organic alcohols_ and acids 26.833333
## 2 Phenylalanine_ tyrosine and tryptophan biosynthesis 28.411765
## 3 Pyruvate metabolism 60.375000
## 4 Protein export_ secretion_ and sorting 6.066388
## 5 Cell division 12.710526
## 6 Transcription factors 6.355263
## PercentageInModule
## 1 28.571429
## 2 14.285714
## 3 14.285714
## 4 3.947368
## 5 5.263158
## 6 2.631579

# melt then combine into Enriched Roles
mr.melt <- melt(MR, measure.vars = "Main Role")
sr.melt <- melt(SR, measure.vars = "Subrole")
head(mr.melt)

## dir Treatment Ratio PercentageInModule
## 1 Increase HL-48 Glucose\nCompetition 4.364458 42.857143
## 2 Increase HL-48 Xylose\nCommensalism 5.105047 9.210526
## 3 Increase HL-48 Xylose\nCommensalism 3.607041 7.894737
## 4 Increase HL-48 Xylose\nCommensalism 3.681670 15.789474
## 5 Increase HL-48 Xylose\nCommensalism 9.885965 5.263158
## 6 Increase HL-58 Glucose\nCompetition 1.827526 6.319703
## variable value
## 1 Main Role Transport and binding proteins
## 2 Main Role Cell structure_ growth_ and death
## 3 Main Role Energy metabolism
## 4 Main Role Translation
## 5 Main Role Transcription
## 6 Main Role Intracellular trafficking_ assembly_ and processing

head(sr.melt)

## dir Treatment Ratio PercentageInModule
## 1 Increase HL-48 Glucose\nCompetition 26.833333 28.571429
## 2 Increase HL-48 Glucose\nCompetition 28.411765 14.285714
## 3 Increase HL-48 Glucose\nCompetition 60.375000 14.285714
## 4 Increase HL-48 Xylose\nCommensalism 6.066388 3.947368
## 5 Increase HL-48 Xylose\nCommensalism 12.710526 5.263158
## 6 Increase HL-48 Xylose\nCommensalism 6.355263 2.631579
## variable value
## 1 Subrole Carbohydrates_ organic alcohols_ and acids
## 2 Subrole Phenylalanine_ tyrosine and tryptophan biosynthesis
## 3 Subrole Pyruvate metabolism
## 4 Subrole Protein export_ secretion_ and sorting
## 5 Subrole Cell division
## 6 Subrole Transcription factors

el.melt <- rbind(mr.melt, sr.melt)

# rename treatments
el.melt$Treatment <- el.melt$Treatment %>% factor(labels =
 c("HL-48 \nGlucose\nCompetition", "HL-48 \nXylose\nCommensalism", "HL-58 \nGlucose\nCompetition"))
# rename treatments (without mentioning sugar type)
#el.melt$Treatment <- el.melt$Treatment %>% factor(labels = c("HL-48 \nCompetition", "HL-48 \nCommensalism", "HL-58 \nCompetition"))

# reorder levels
el.melt$Treatment <- factor(el.melt$Treatment, levels(el.melt$Treatment)[c(1,3,2)])


# Remove underscore
el.melt$value <- gsub("_", "", fixed = T, x = el.melt$value)
# Manually edit y-axis elements in this graph. Easy way to change text text
# pico(el.melt$value) # Get text
# el.melt$value <- c("the string"")

# Also remove the unidentified item
el.melt <- el.melt %>% subset(value != "")

g.el.FE <- ggplot(el.melt, aes(x = Treatment, y = value, size = Ratio, fill = PercentageInModule))
g.el.FE + geom_point(shape = 21) + labs(x = "", y = "") + facet_grid(variable~dir, scales = "free_y", space = "free") +
scale_size_continuous(range = c(2, 10)) + scale_fill_viridis(option = "B", begin = .1, end = .9) +
theme(
 #legend.position = c(-0.9, -0.12), legend.direction = "horizontal", legend.box = "vertical", plot.margin = unit(c(10, 5.5, 30, 5.5), "points"),
 legend.position = c(-0.5, -0.10), legend.direction = "horizontal", legend.box = "vertical", plot.margin = unit(c(10, 5.5, 25, 5.5), "points"),
 strip.background = element_blank(), strip.text = element_text(size = 12), legend.spacing.y = unit(0, "points"),
 axis.text.x = element_text(angle = -30, hjust=0, vjust = 1, size = 10)) # angle of 30

ggsave("figures/fig2.pdf", width = 120, height = 120, units = "mm", scale = 1.5)

### Additional FE analysis of data from the three sup. volcano plots

# Input data sets

HL48.diff.coculture.f <- HL48.diff.coculture %>% subset(padj <= 0.05)
HL58.diff.coculture.f <- HL58.diff.coculture %>% subset(padj <= 0.05)
HL58.diff.proxy.f <- HL58.diff.proxy %>% subset(padj <= 0.05)

HL48.diff.coculture.U <- HL48.diff.coculture.f %>% subset(log2FoldChange >= 1)
HL58.diff.coculture.U <- HL58.diff.coculture.f %>% subset(log2FoldChange >= 1)
HL58.diff.proxy.U <- HL58.diff.proxy.f %>% subset(log2FoldChange >= 1)
HL48.diff.coculture.D <- HL48.diff.coculture.f %>% subset(log2FoldChange <= -1)
HL58.diff.coculture.D <- HL58.diff.coculture.f %>% subset(log2FoldChange <= -1)
HL58.diff.proxy.D <- HL58.diff.proxy.f %>% subset(log2FoldChange <= -1)

#prepare input files for FE
HL48.diff.coculture.U.FE <- HL48.diff.coculture.U %>% select(GeneID) %>% data.frame(., ModuleID = "test")
HL58.diff.coculture.U.FE <- HL58.diff.coculture.U %>% select(GeneID) %>% data.frame(., ModuleID = "test2")
HL58.diff.proxy.U.FE <- HL58.diff.proxy.U %>% select(GeneID) %>% data.frame(., ModuleID = "test3")
HL48.diff.coculture.D.FE <- HL48.diff.coculture.D %>% select(GeneID) %>% data.frame(., ModuleID = "test4")
HL58.diff.coculture.D.FE <- HL58.diff.coculture.D %>% select(GeneID) %>% data.frame(., ModuleID = "test5")
HL58.diff.proxy.D.FE <- HL58.diff.proxy.D %>% select(GeneID) %>% data.frame(., ModuleID = "test6")

# main role
# Up
HL48.diff.coculture.U.FE.MR <- mainroleeModuleEnrichment(HL48.diff.coculture.U.FE, an.48)
HL48.diff.coculture.U.FE.MR$Treatment <- 'HL-48 Coculture'
HL48.diff.coculture.U.FE.MR$dir <- "Increase"
HL58.diff.coculture.U.FE.MR <- mainroleeModuleEnrichment(HL58.diff.coculture.U.FE, an.58)
HL58.diff.coculture.U.FE.MR$Treatment <- 'HL-58 Coculture'
HL58.diff.coculture.U.FE.MR$dir <- "Increase"
HL58.diff.proxy.U.FE.MR <- mainroleeModuleEnrichment(HL58.diff.proxy.U.FE, an.58)
HL58.diff.proxy.U.FE.MR$Treatment <- 'HL-58 Proxy'
HL58.diff.proxy.U.FE.MR$dir <- "Increase"
# down
HL48.diff.coculture.D.FE.MR <- mainroleeModuleEnrichment(HL48.diff.coculture.D.FE, an.48)
#HL48.diff.coculture.D.FE.MR$Treatment <- 'HL48 Coculture' # empty
#HL48.diff.coculture.D.FE.MR$dir <- "Decrease" # empty
HL58.diff.coculture.D.FE.MR <- mainroleeModuleEnrichment(HL58.diff.coculture.D.FE, an.58)
HL58.diff.coculture.D.FE.MR$Treatment <- 'HL-58 Coculture'
HL58.diff.coculture.D.FE.MR$dir <- "Decrease"
HL58.diff.proxy.D.FE.MR <- mainroleeModuleEnrichment(HL58.diff.proxy.D.FE, an.58)
HL58.diff.proxy.D.FE.MR$Treatment <- 'HL-58 Proxy'
HL58.diff.proxy.D.FE.MR$dir <- "Decrease"


#pull it together
new.MR.FE <- rbind(HL48.diff.coculture.U.FE.MR, HL58.diff.coculture.U.FE.MR, HL58.diff.proxy.U.FE.MR,
 HL48.diff.coculture.D.FE.MR, HL58.diff.coculture.D.FE.MR, HL58.diff.proxy.D.FE.MR)
#kable(new.MR.FE) #caption = "Functional enrichment of main role gene categories")

# Sub role
# Up
HL48.diff.coculture.U.FE.SR <- subroleModuleEnrichment(HL48.diff.coculture.U.FE, an.48)
HL48.diff.coculture.U.FE.SR$Treatment <- 'HL-48 Coculture'
HL48.diff.coculture.U.FE.SR$dir <- "Increase"
HL58.diff.coculture.U.FE.SR <- subroleModuleEnrichment(HL58.diff.coculture.U.FE, an.58)
HL58.diff.coculture.U.FE.SR$Treatment <- 'HL-58 Coculture'
HL58.diff.coculture.U.FE.SR$dir <- "Increase"
HL58.diff.proxy.U.FE.SR <- subroleModuleEnrichment(HL58.diff.proxy.U.FE, an.58)
HL58.diff.proxy.U.FE.SR$Treatment <- 'HL-58 Proxy'
HL58.diff.proxy.U.FE.SR$dir <- "Increase"
# down
HL48.diff.coculture.D.FE.SR <- subroleModuleEnrichment(HL48.diff.coculture.D.FE, an.48)
HL48.diff.coculture.D.FE.SR$Treatment <- 'HL-48 Coculture'
HL48.diff.coculture.D.FE.SR$dir <- "Decrease"
HL58.diff.coculture.D.FE.SR <- subroleModuleEnrichment(HL58.diff.coculture.D.FE, an.58)
HL58.diff.coculture.D.FE.SR$Treatment <- 'HL-58 Coculture'
HL58.diff.coculture.D.FE.SR$dir <- "Decrease"
HL58.diff.proxy.D.FE.SR <- subroleModuleEnrichment(HL58.diff.proxy.D.FE, an.58)
HL58.diff.proxy.D.FE.SR$Treatment <- 'HL-58 Proxy'
HL58.diff.proxy.D.FE.SR$dir <- "Decrease"


#pull it together
new.SR.FE <- rbind(HL48.diff.coculture.U.FE.SR, HL58.diff.coculture.U.FE.SR, HL58.diff.proxy.U.FE.SR,
 HL48.diff.coculture.D.FE.SR, HL58.diff.coculture.D.FE.SR, HL58.diff.proxy.D.FE.SR)
#kable(new.SR.FE) #caption = "Functional enrichment of sub-role gene categories")

new.MR <- data.frame(dir = new.MR.FE$dir, Treatment = new.MR.FE$Treatment, Main_role = new.MR.FE$Main_Role,
 Ratio = as.numeric(new.MR.FE$Ratio), PercentageInModule = 100*(as.numeric(new.MR.FE$PercentageInModule)))

new.SR <- data.frame(dir = new.SR.FE$dir, Treatment = new.SR.FE$Treatment, Subrole = new.SR.FE$Subrole,
 Ratio = as.numeric(new.SR.FE$Ratio), PercentageInModule = 100*(as.numeric(new.SR.FE$PercentageInModule)))

names(new.MR)[3] <- "Main Role"
names(new.SR)[3] <- "Subrole"

head(new.MR)

## dir Treatment Main Role
## 1 Increase HL-48 Coculture Carbohydrate metabolism
## 2 Increase HL-58 Coculture Carbohydrate metabolism
## 3 Increase HL-58 Proxy Xenobiotics biodegradation and metabolism
## 4 Increase HL-58 Proxy Fatty acid and lipid metabolism
## 5 Decrease HL-58 Coculture Xenobiotics biodegradation and metabolism
## 6 Decrease HL-58 Coculture
## Ratio PercentageInModule
## 1 2.396173 6.024096
## 2 1.847878 4.291845
## 3 5.740741 1.481481
## 4 1.845238 3.333333
## 5 5.391304 1.391304
## 6 1.328772 46.086957

head(new.SR)

## dir Treatment Subrole
## 1 Increase HL-48 Coculture Polysacharide and lipopolysaccharide metabolism
## 2 Increase HL-48 Coculture Invasion response
## 3 Increase HL-48 Coculture Glycolysis / Gluconeogenesis
## 4 Increase HL-48 Coculture Pyruvate metabolism
## 5 Increase HL-48 Coculture Butanoate metabolism
## 6 Increase HL-48 Coculture Anions
## Ratio PercentageInModule
## 1 2.586345 3.212851
## 2 5.222428 2.008032
## 3 4.849398 2.008032
## 4 5.091867 1.204819
## 5 10.183735 1.204819
## 6 5.657631 4.016064

# melt then combine into Enriched Roles
new.mr.melt <- melt(new.MR, measure.vars = "Main Role")
new.sr.melt <- melt(new.SR, measure.vars = "Subrole")
head(new.mr.melt)

## dir Treatment Ratio PercentageInModule variable
## 1 Increase HL-48 Coculture 2.396173 6.024096 Main Role
## 2 Increase HL-58 Coculture 1.847878 4.291845 Main Role
## 3 Increase HL-58 Proxy 5.740741 1.481481 Main Role
## 4 Increase HL-58 Proxy 1.845238 3.333333 Main Role
## 5 Decrease HL-58 Coculture 5.391304 1.391304 Main Role
## 6 Decrease HL-58 Coculture 1.328772 46.086957 Main Role
## value
## 1 Carbohydrate metabolism
## 2 Carbohydrate metabolism
## 3 Xenobiotics biodegradation and metabolism
## 4 Fatty acid and lipid metabolism
## 5 Xenobiotics biodegradation and metabolism
## 6

head(new.sr.melt)

## dir Treatment Ratio PercentageInModule variable
## 1 Increase HL-48 Coculture 2.586345 3.212851 Subrole
## 2 Increase HL-48 Coculture 5.222428 2.008032 Subrole
## 3 Increase HL-48 Coculture 4.849398 2.008032 Subrole
## 4 Increase HL-48 Coculture 5.091867 1.204819 Subrole
## 5 Increase HL-48 Coculture 10.183735 1.204819 Subrole
## 6 Increase HL-48 Coculture 5.657631 4.016064 Subrole
## value
## 1 Polysacharide and lipopolysaccharide metabolism
## 2 Invasion response
## 3 Glycolysis / Gluconeogenesis
## 4 Pyruvate metabolism
## 5 Butanoate metabolism
## 6 Anions

new.el.melt <- rbind(new.mr.melt, new.sr.melt)

# rename treatments. (Maybe not needed based on my new names for 'modules')
# new.el.melt$Treatment <- el.melt$Treatment %>% factor(labels = c("HL48 \nGlucose\nCompetition", "HL48 \nXylose\nCommensalism", "HL58 \nGlucose\nCompetition"))

# Remove underscore
new.el.melt$value <- gsub("_", "", fixed = T, x = new.el.melt$value)
# Manually edit y-axis elements in this graph. Easy way to change text text
# pico(el.melt$value) # Get text
# el.melt$value <- c("the string"")

# Also remove the unidentified item
new.el.melt <- new.el.melt %>% subset(value != "")

g.new.el.FE <- ggplot(new.el.melt, aes(x = Treatment, y = value, size = Ratio, fill = PercentageInModule))
g.new.el.FE + geom_point(shape = 21) + labs(x = "", y = "") + facet_grid(variable~dir, scales = "free_y", space = "free") +
scale_size_continuous(range = c(2, 10)) + scale_fill_viridis(option = "B", begin = .1, end = .9) +
theme(
 #legend.position = c(-0.9, -0.12), legend.direction = "horizontal", legend.box = "vertical", plot.margin = unit(c(10, 5.5, 30, 5.5), "points"),
 legend.position = c(-0.5, -0.10), legend.direction = "horizontal", legend.box = "vertical", plot.margin = unit(c(10, 10, 35, 5.5), "points"),
 strip.background = element_blank(), strip.text = element_text(size = 12), legend.spacing.y = unit(0, "points"),
 axis.text.x = element_text(angle = -20, hjust=0, vjust = 1, size = 10))


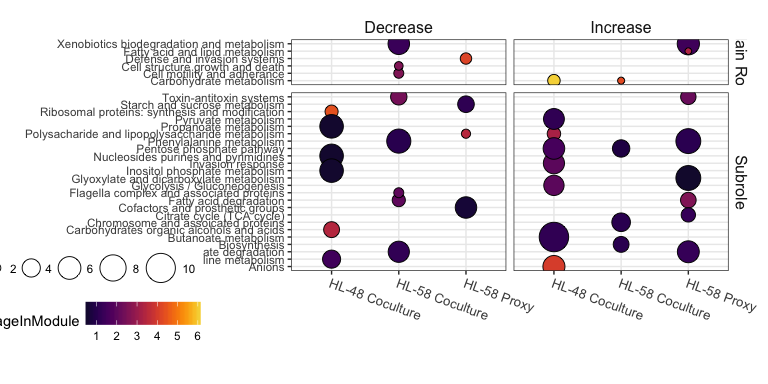


ggsave("figures/sub-FE-new.pdf", width = 114, height = 140, units = "mm", scale = 1.5)

#Save and exit.
knitr::knit_exit(F)

FALSE
