# Supplementary material for: Phenotypic responses to interspecies competition and commensalism in a naturally-derived microbial co-culture
Source: Sci Rep. 2018 Jan 10;8:297. doi: 10.1038/s41598-017-18630-1 (PMC5762899; doi:10.1038/s41598-017-18630-1)
Supplement: Supplementary file 2 — Supplementary data and results [file 41598_2017_18630_MOESM2_ESM.doc]

**Supplemental information for:** Phenotypic responses to interspecies competition and commensalism in a naturally-derived microbial co-culture

Nymul Khan^1^, Yukari Maezato^1^, Ryan S. McClure^1^, Colin J. Brislawn^1^, Jennifer M. Mobberley^1^, Nancy Isern^2^, William B. Chrisler^1,2^, Lye Meng Markillie^2^, Brett M. Barney^3^, Hyun-Seob Song^1^, William C. Nelson^1^, Hans C. Bernstein^1,4^*

^1^Biological Sciences Division, Pacific Northwest National Laboratory, Richland, WA, USA; ^2^ Environmental Molecular Sciences Laboratory, Pacific Northwest National Laboratory, Richland, Washington, USA; ^3^Department of Bioproducts and Biosystems Engineering, University of Minnesota, St. Paul, MN 55108; ^4^The Gene and Linda Voiland School of Chemical Engineering and Bioengineering, Washington State University, Pullman, WA, USA

**** Correspondence:***Hans C. Bernstein, Biological Sciences Division, Pacific Northwest National Laboratory, P.O. Box 999, MS-IN: J4-18, Richland, WA 99352 ([Hans.Bernstein@pnnl.gov](mailto:Hans.Bernstein@pnnl.gov));


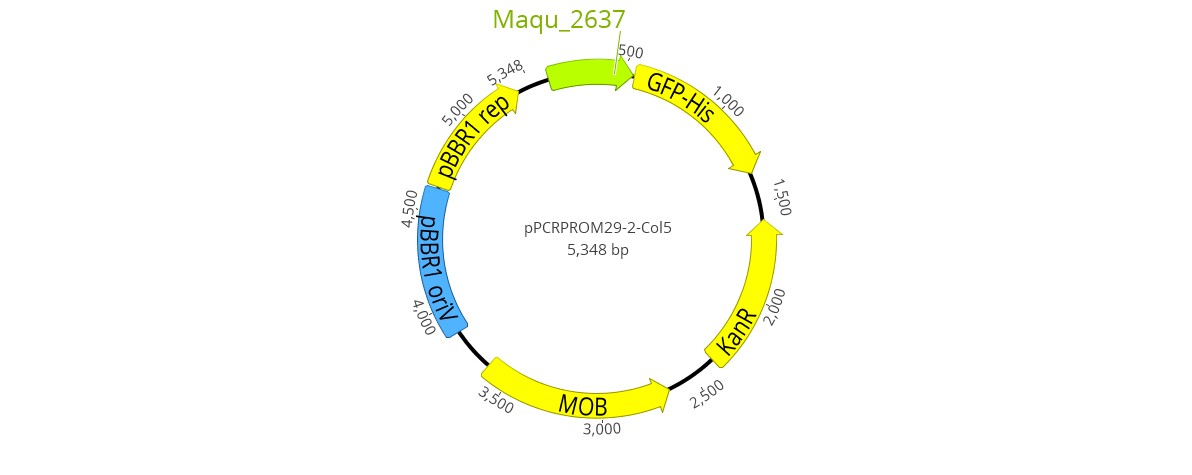
**Figure S1.** Plasmid pPCRProm29-Col5: A broad-host-range plasmid based on pBBR1MCS2 where the Plac-lacZa cassette has been replaced with Maqu_2637-GFP-His cassette for fluorescent expression.

Construction of a plasmid for protein expression and to serve as a reporter in *Marinobacter* and related species was based on results from a whole transcriptome shotgun sequencing (RNA-Seq) study of the genes from *Marinobacter aquaeolei* VT8. As a result of these studies, we selected several target genes that were highly transcribed and targeted the genomic regions upstream of these genes for cloning to construct an initial reporter vector. The promoter region for Maqu_2637 was cloned from *M. aquaeolei* VT8 using primer BBP2010 (5’NNNAAGCT TCATATGAAA CGTTCCTATA AGTTTCCAAG TTTTAATGAC CTTG3’), which contains a *HindIII* site and an *NdeI* site at the ATG start codon in place of the Maqu_2637 gene and primer BBP2011 (5’CTGAAGGG AACCTGTAAG ACAGCTGG3’) which is upstream of a native *KpnI* site upstream of the promoter region. The promoter region was ligated into the *HindIII* and *KpnI* sites of plasmid pBB053 (Lenneman reference) and sequenced to confirm no mistakes. The plasmid backbone for the broad host range vector is based on the pBBR1MCS-2 plasmid, which was modified by a series of steps to remove *NcoI*, *NsiI* and *KpnI* sites, while also incorporating a 38 bp sequence (5’CTTAACGT GAGTTTTCGT TCCACTGAGC GTCAGACCCC3’) which protects the plasmid from potential transposon uptake from the *Escherichia coli* WM3064 host strain during conjugations, resulting in plasmid pBB109. The gene segment containing the Maqu_2637 promoter region was shuttled into pBB109 vector by digesting both with *KpnI* and *NdeI*, then ligating the construct. The segment containing a restriction enzyme optimized version of the gene for GFP (plasmid pGFPUV with internal *NcoI* and *NdeI* sites removed by silent mutations, [pGFPUV obtained from Clontech, Mountain View, CA]) flanked by an *NdeI* site at the start codon and an *EcoRI* site after the stop codon, was then inserted behind the promoter to result in the vector pPCRPROM29. The sequence of the final pPCRPROM29 vector is available as well as the vector from Addgene ([www.addgene.org](http://www.addgene.org/)). The GFP from this construct contains an N-terminal polyhistidine tag for simple protein isolation to determine expression yield of the GFP. The plasmid has been successfully tested in *M. aquaeolei* VT8 and *Pyschrobacter cryohalolentis* K5.


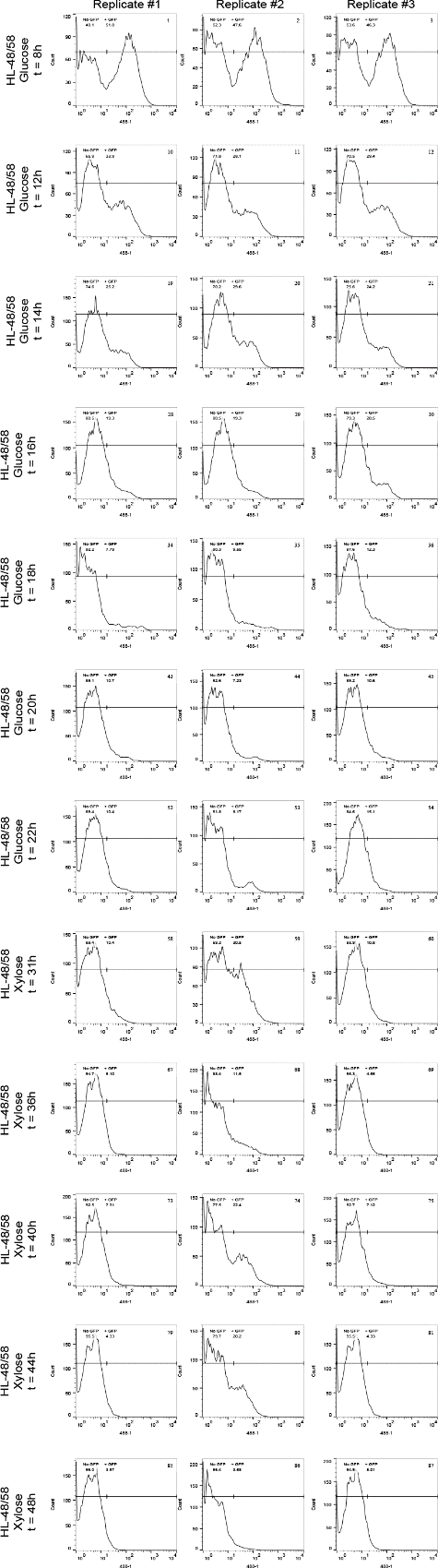
**Figure S2**. Flow cytometry histograms of GFP expression. GFP signal from cells suspensions was measured in the FL1 or GFP channel. The Mean Fluorescence Intensity (MFI) of *Marinobacter sp. HL-58* (+GFP) and *Halomonas sp. HL-48* (-GFP), cell populations was reported on the x-axis. Gates were drawn to discriminate the ratio of the two populations.


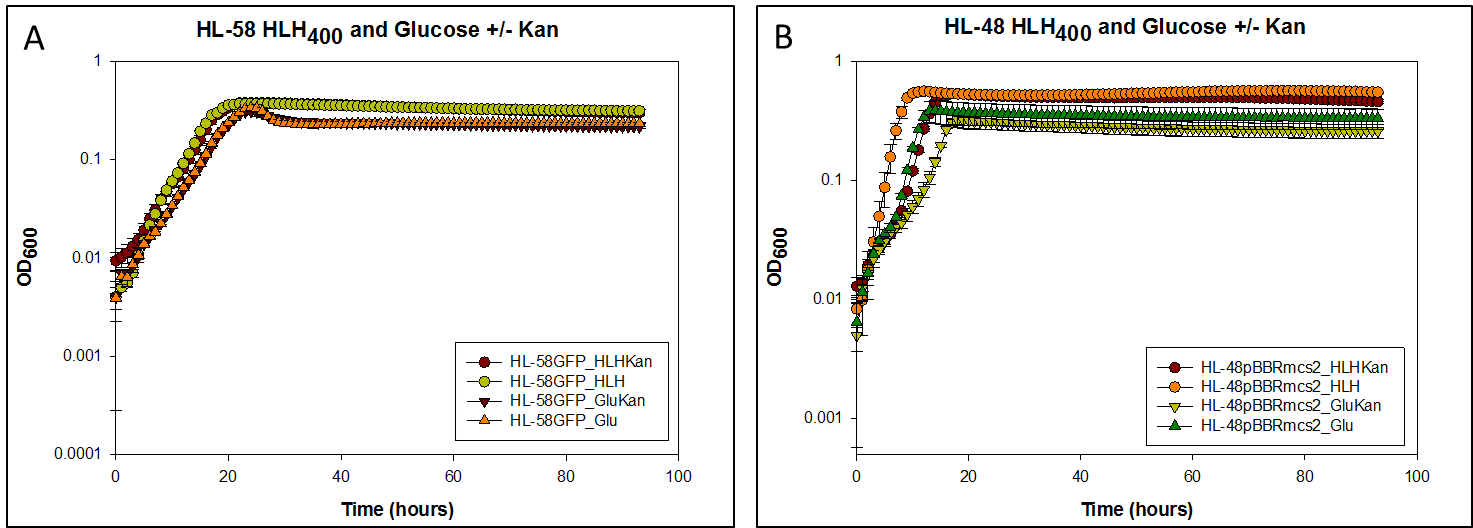


**Figure S3**. Growth curves collected for the experimental strains compared with and without 50 µg ml^-1^ kanamycin. **A)** *Marinobacter* sp. HL-58 pPCRPROM29-2-GFP and **B)** *Halomonas* sp. HL-48 pBBR-MCS2. The experiments were performed in the experimental Hot Lake Heterotrophic medium containing 0.1% yeast extract (HLH) and defined medium supplemented with 5mM glucose (Glu). Each data point represents the mean from 3 biological replicates and error bars represent ± 1 standard deviation.
